# Performance and Applications of Polymer Fiber Rubber-Reinforced Concrete in Civil Engineering: A State-of-the-Art Review

**DOI:** 10.3390/polym17070970

**Published:** 2025-04-02

**Authors:** Jie Liu, Wantao Xu, Guansheng Li, Bing Chen, Yi Xiao, Huiping Huang, Juanjuan Chen

**Affiliations:** 1 School of Civil Engineering and Architecture, Wuhan Institute of Technology, Wuhan 430074, China; 21090101@wit.edu.cn (J.L.); 22404010011@stu.wit.edu.cn (W.X.); 22404010014@stu.wit.edu.cn (G.L.); 22404010119@stu.wit.edu.cn (B.C.); 22404010091@stu.wit.edu.cn (Y.X.); 2 Hubei Provincial Engineering Research Center for Green Civil Engineering Materials and Structures, Wuhan Institute of Technology, Wuhan 430074, China; 3 Key Laboratory of Concrete and Prestressed Concrete Structures of Ministry of Education, National Engineering Research Center for Prestressing Technology, School of Civil Engineering, Southeast University, Nanjing 211189, China; 230228342@seu.edu.cn; 4 School of Architecture Engineering, College of Post and Telecommunication of WIT, Wuhan 430073, China

**Keywords:** polymer fiber, rubber, concrete, mechanical properties, civil engineering

## Abstract

Polymer fiber rubber-reinforced concrete (PFRRC) represents a high-performance composite material that synergistically integrates the energy absorption of rubber concrete (RC) and the crack resistance of polymer fiber-reinforced concrete (PFRC). This review systematically evaluates the mechanical and durability properties of PFRRC, emphasizing its potential to overcome the intrinsic brittleness of conventional concrete while enhancing structural resilience. Experimental results indicate that PFRRC exhibits significant improvements in compressive, tensile, and flexural strength, with increases of up to 29%, 38%, and 66%, respectively, compared to RC. Furthermore, it demonstrates exceptional impact resistance, with energy absorption capabilities up to 10 times greater than that of ordinary concrete. The hybrid composite also demonstrates enhanced durability, including reduced chloride ion penetration (24.5% lower diffusion coefficient) and improved freeze–thaw resistance. However, challenges remain in optimizing rubber–polymer interactions, fiber hybridization ratios, and performance under extreme conditions. By addressing these limitations, PFRRC holds transformative potential for sustainable infrastructure, particularly in road engineering, seismic-resistant structures, and protective systems.

## 1. Introduction

Concrete, a cementitious material, has a relatively low inherent tensile strength and limited deformation capacity, showing pronounced brittleness. Conventional concrete often fails to meet key performance criteria, like fatigue strength, corrosion resistance, wear resistance, tensile strength, deformation adaptability, shear strength, post-crack load-bearing capacity, and toughness. To overcome these limitations, enhancing concrete’s overall performance requires not only improving its basic properties but also developing high-performance composites to broaden its application range.

Rubber concrete (RC) is an innovative construction material combining crumb rubber (CR) with conventional concrete. Replacing natural aggregates with rubber ones reduces concrete’s brittleness, enabling it to absorb more energy under tension and compression, enhancing ductility and plastic failure capacity [1,2,3]. Its dynamic performance improves notably, with an increased damping ratio [4,5,6], better resistance to cracking [7,8], enhanced freeze–thaw resistance [9,10], and greater resistance to hydrochloric acid [11,12]. However, adding rubber poses challenges to the bonding within the cement paste. When rubber particles do not bond well, the mechanical and durability properties of the cement paste deteriorate [13,14,15,16]. RC’s compressive strength, elastic modulus, splitting tensile strength, and flexural strength are much lower than those of conventional concrete. As the rubber content rises, strength drops further, limiting its use in structural building [17,18,19,20,21].

Polymer fiber-reinforced concrete (PFRC) is another innovative composite drawing researchers’ attention. Adding an appropriate amount of polymer fibers to concrete markedly boosts its mechanical properties, such as compressive and tensile strength [22,23,24,25], flexural toughness [24,26,27], shrinkage resistance [24,28], and durability [29,30,31]. This enhanced performance stems mainly from fiber–cementitious matrix compatibility and stress transfer via surface adhesion. Strong interfacial properties ensure efficient energy transfer from the matrix to the fibers, improving the composite’s mechanicals [32]. But when polymer fiber content exceeds a certain level, it can harm concrete, like reducing strength [33,34].

The high-performance composite polymer fiber rubber-reinforced concrete (PFRRC) incorporates polymer fibers and rubber particles into conventional concrete to address PFRC’s and RC’s limitations while optimizing their advantages. Studies suggest that PFRRC has promising properties for structural use [35,36,37,38], enhancing structural safety and service life, and aiding green building, sustainable development, and disaster resilience [39]. However, a review of PFRRC literature shows ongoing challenges. For example, methods for evaluating rubber reinforcement are underdeveloped, and the performance enhancement of different fiber types in the cement matrix is debated. Research on PFRRC’s material properties is limited, and its engineering applications are scarce. Thus, it is crucial to systematically review current high-quality PFRRC research, provide data for performance studies, and offer insights for engineering applications.

## 2. RC

### 2.1. Mechanical Properties of RC

#### 2.1.1. Micromorphology Analysis

Scanning electron microscopy (SEM) is a vital technique for examining the internal microstructure of materials. It is used to analyze the composition of the pore structure in the cement matrix, the morphology of the matrix, and the characteristics of the hydration products.

**Figure 1:**
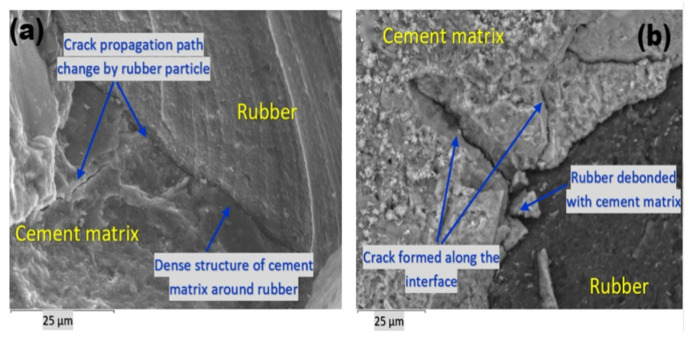
Microstructure of concrete with and without rubber (**a**) SEM microstructure image of the specimen without RC; (**b**) SEM microstructure image of the specimen with 10% RC [22].

Dong et al. [40] revealed that rubber particles promote gas entrapment during mixing, increasing interfacial porosity and reducing the composite density. The resulting transition zones exhibit sparse hydration products, creating weak interfacial bonds that degrade mechanical performance proportionally with rubber content. Silva et al. [41] corroborated this phenomenon, identifying increased micropore formation at 30% rubber replacement that induces transition zone cracking (Figure 1). However, complete rubber fiber encapsulation by cement slurry can reduce porosity and enhance density, suggesting that optimal dispersion mitigates negative effects. Reda et al. [42] observed that there was a weak bond between the tire rubber particles and the cement matrix based on crack analysis. However, at the sites where the rubber particles were extracted, indentations were formed on the cement matrix, thus improving bond strength. Additionally, the microcracks surrounding the tire particles and the internal tensile cracking of the rubber particles demonstrate that the rubber particles enhance the toughness of RC and improve its impact resistance and fracture toughness.

Although the incorporation of rubber particles into concrete may lead to issues, such as increased porosity and reduced density, they enhance the toughness and energy absorption capacity of RC through mechanisms including microcrack formation and internal tensile cracking. Particularly during impact and fracture events, they effectively absorb energy and significantly improve toughness.

#### 2.1.2. Compressive Properties

In the construction industry, the compressive strength of concrete is regarded as its most crucial property. Before using any concrete mix in construction, it must meet the required compressive—strength standards for its intended application. The literature commonly agrees that as the proportion of rubber in concrete increases, the reduction in compressive strength becomes more significant.

According to the study on mechanical properties of RC by Dong et al. [40], 1–2 mm rubber aggregate was obtained by mechanical crushing and screening of waste tires, and replaced the fine aggregate in concrete at a volume ratio of 10% and 20%, respectively. The study showed that the compressive strength of concrete decreased significantly with the increase in rubber content. Mohseni et al. [43] used 10–15 mm rubber aggregate to replace coarse aggregate and tested the compressive strength of RC samples at 7 days and 28 days of age. The results showed that the compressive strength gradually decreased with the increase in rubber content, further verifying the negative correlation between rubber content and concrete strength.

**Figure 2:**
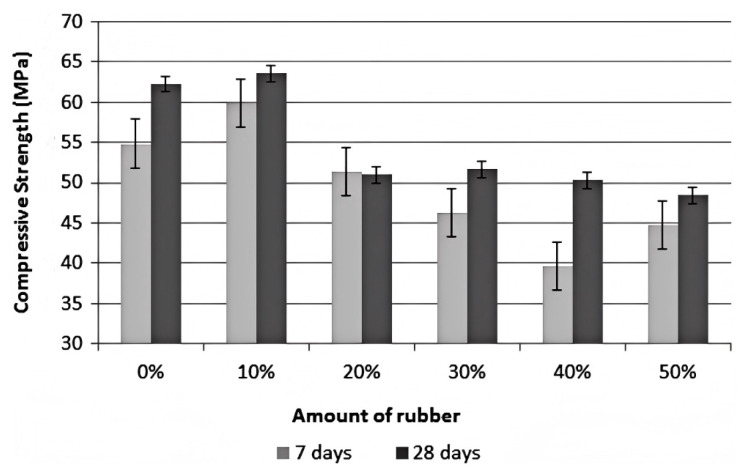
Relationship between compressive strength and rubber content [41].

Furthermore, Silva et al. [41] used rubber aggregates sized between 1.18 and 2.36 mm to replace natural sand in their research on rubber tactile paving blocks. The experimental findings (Figure 2) show that as the rubber content rises, the compressive strength of concrete steadily decreases. When the rubber content reaches 50%, the 28-day compressive strength can drop by as much as 22%. Although Stallings et al. [44]’s experimental results also showed a decrease in compressive strength with increasing rubber content, their study found that in fine aggregate samples, the reduction in compressive strength is less severe. This indicates that the impact of fine aggregate replacement on concrete strength is relatively minor. Finally, Azunna et al. [21] comprehensively summarized the effects of rubber replacement on the strength of both coarse and fine aggregates in concrete. As presented in Table 1, the compressive strength of concrete is higher when fine aggregate is replaced by CR than when coarse aggregate is replaced, further emphasizing the different effects of aggregate type on concrete strength.

Therefore, regardless of whether coarse or fine aggregate is replaced, the compressive strength declines as the rubber substitution rate increases, exhibiting a generally negative correlation; the replacement of fine aggregate with CR typically results in a lesser reduction in compressive strength compared to the replacement of coarse aggregate.

#### 2.1.3. Splitting Tensile Strength

The splitting tensile strength of RC is a crucial property. It not only determines the load at which concrete components crack but can also be used to assess structural component designs for crack prevention and failure avoidance. The literature generally agrees that an increase in rubber content reduces the splitting tensile strength of RC.

**Figure 3:**
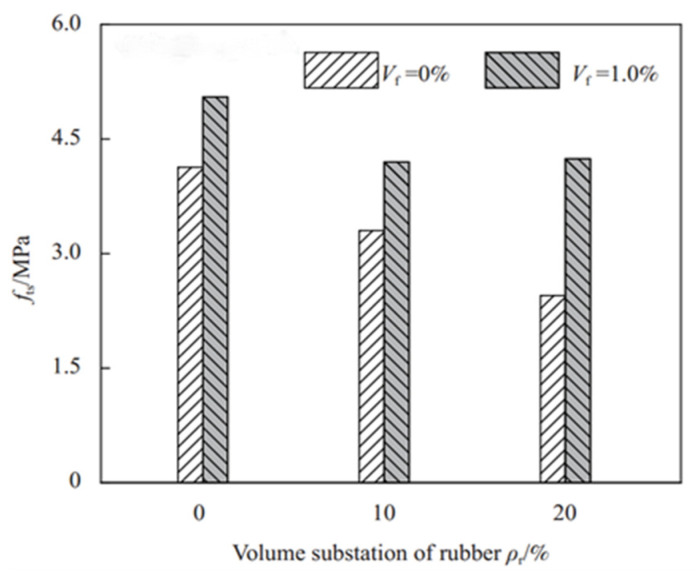
Effect of different rubber content on tensile strength [40].

Dong et al. [40] found that adding rubber particles to RC decreased the splitting tensile strength by over 15% in specimens with 10% rubber particles. As the rubber content rose, tensile strength continued to drop, as shown in Figure 3. Li et al. [45] studied the impact of waste tire rubber fibers and chips on concrete’s splitting tensile strength. The results showed that concrete with rubber chips or fibers had lower strength than the control, with relevant data in Table 2. Wang et al. [22] replaced fine aggregate with waste tire rubber aggregates of different sizes. When 20% of sand was replaced, splitting tensile strength decreased by about 26%, and further increases in rubber content led to a further strength reduction. This supports the negative impact of rubber content on RC’s splitting tensile strength. Eldin et al. [46] replaced coarse and fine aggregates with rubber. When coarse aggregate replacement was 100%, 28-day strength decreased by 75%, and for 100% fine aggregate replacement, it decreased by 49%, suggesting that fine aggregate replacement has a relatively minor effect on RC strength.

In conclusion, increasing the rubber content reduces the splitting tensile strength of RC, whether replacing coarse or fine aggregate, with fine aggregate replacement having a smaller impact. This aligns with findings on compressive strength, highlighting the effects of aggregate type and content on RC performance.

#### 2.1.4. Flexural Strength

The flexural strength of concrete is one of its crucial properties. Current research commonly shows a negative correlation between the flexural strength of RC and rubber content; that is, as rubber content rises, flexural strength gradually decreases.

Silva et al. [41] used different—sized and—proportioned rubber to replace fine aggregate (natural sand) in studying recycled tire—based RC tactile pavement bricks. Their results indicated that flexural strength dropped as rubber content increased. When 50% of the aggregate was replaced by rubber, flexural strength decreased by about 32%. Dong et al. [40] replaced 10% and 20% of the fine aggregate volume in RC with 1–2 mm rubber aggregates. The results showed RC’s failure mode was brittle fracture, and flexural strength decreased with more rubber, in line with Fabiana et al.’s findings.

**Figure 4:**
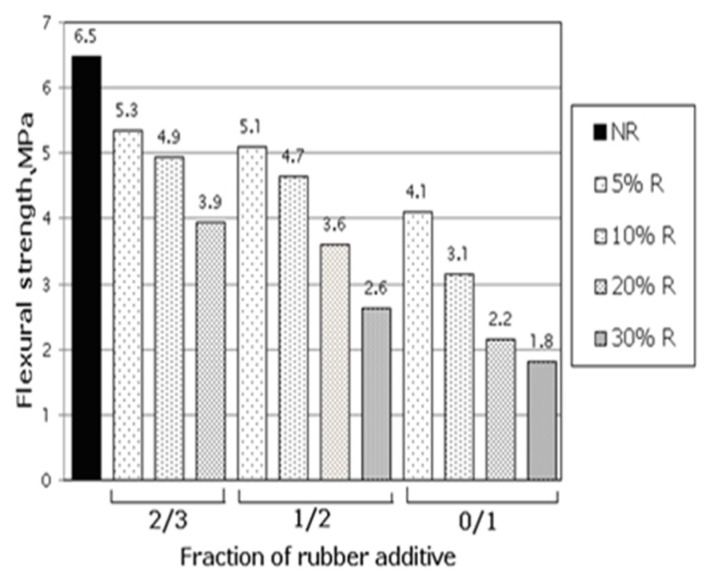
Effect of different rubber contents in each particle size group on flexural strength [48].

Subsequent studies by Khatib et al. [47] confirmed this, finding that flexural strength decreased with larger rubber aggregate size and more content. Skripkinunas et al. [48] obtained similar results with differently sized rubber aggregates (Figure 4). Aliabdo et al. [49] compared traditional and RC flexural strength, finding up to an 84% reduction in flexural strength with more rubber particles.

In conclusion, the flexural strength of RC declines as the rubber incorporation rate rises. Significantly, replacing fine aggregates has a less harmful effect on flexural strength than replacing coarse aggregates, consistent with previous findings on compressive and tensile strengths.

#### 2.1.5. Toughness

Toughness refers to the capacity of concrete to undergo deformation without failure when subjected to external forces, including tension, bending, and torsion. Recent studies on RC suggest that the incorporation of rubber enhances the material’s toughness.

Liu et al. [50] investigated the impact of substituting fine aggregates with rubber particles on the fatigue life of concrete. Using a three-point fatigue bending test, the results revealed that, at a constant stress level, an increased rubber content correlated with a longer fatigue life of concrete. Consequently, RC demonstrates superior toughness and resistance to deformation compared to conventional concrete. Reda et al. [42] examined the influence of rubber content in RC on its fracture toughness. The results indicated that as the rubber content increased, fracture toughness improved, and only began to decrease once the content exceeded 75%, suggesting that RC’s toughness was notably enhanced at lower rubber content levels (refer to Table 3). Li et al. [45] also demonstrated that the incorporation of waste tire rubber markedly enhanced concrete toughness. RC exhibits a superior energy absorption capacity and enhanced resistance to crack propagation.

In conclusion, the findings of multiple studies consistently demonstrate that the incorporation of rubber notably enhances concrete toughness, particularly with regard to energy absorption and crack resistance.

#### 2.1.6. Impact Resistance

The impact resistance of concrete is crucial for the safety and structural integrity of engineering structures. Recent research shows that adding rubber significantly boosts the impact resistance of RC.

Aly et al. [51] studied how replacing aggregates with rubber particles affects concrete’s impact resistance. The experimental results showed that as the waste–rubber content rose, impact resistance improved, and the crack pattern shifted from a single large crack, indicating enhanced ductility and impact resistance. Iqbal et al. [52] found that more rubber increased RC’s impact—energy absorption. Tayeb et al. [53] noted a significant boost in RC’s impact resistance when the rubber content reached 20%, maximizing bending load and fracture energy. However, Reda et al. [42] found that over 50% rubber replacement reduced RC’s impact resistance and energy absorption. Atahan et al. [54] showed through dynamic impact tests that energy absorption increased with a higher rubber content, peaking at an 80% replacement ratio. Aliabdo et al. [49] demonstrated that while RC’s impact strength dropped with more rubber, the number of crack–propagation impacts rose, meaning higher energy absorption.

In conclusion, rubber significantly enhances concrete’s impact resistance and energy—absorption capacity. But choosing the right rubber-incorporation rate is key to maintain both impact resistance and strength.

### 2.2. Durability Performance of RC

#### 2.2.1. Resistance to Chloride Ion Penetration

Although chloride ions do not directly damage the concrete matrix, they can cause steel corrosion via the depassivation effect, endangering concrete’s durability. Thus, concrete’s resistance to chloride ion penetration is a key factor in evaluating its long-term durability.

**Figure 5:**
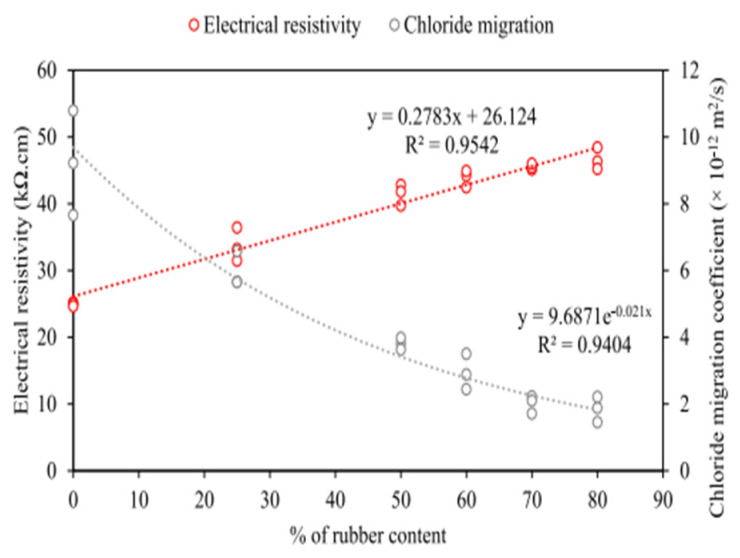
Resistivity and chloride ion migration coefficient of mixtures with different dosages [43].

Mohseni et al. [43] compared chloride ion penetration resistance of rubber-modified and regular mixtures (Figure 5). The chloride ion migration coefficients of RC specimens ranged from 9.22 × 10^−12^ m^2^/s to 1.85 × 10^−12^ m^2^/s, generally decreasing as rubber content rose. This implies that rubber helps form impermeable voids, enhancing chloride resistance. Bravo et al. [55] studied chloride diffusion coefficients with non-steady state migration tests. Cryogenically ground rubber aggregate replacing fine aggregates notably reduced the coefficient compared to the control. Gupta et al. [56] examined chloride ion diffusion coefficients of rubber powder concrete (20% sand replaced) and hybrid RC (10% sand by rubber powder, 25% by rubber fibers). The results showed reductions of 24.5% and 21.81% respectively, as the fine rubber powder was hydrophobic and fibers increased the matrix curvature, delaying chloride ingress. Gesoğlu et al. [57] explored the effects of rubber content and curing duration. Chloride penetration depth rose with more rubber, especially at high water-to-binder ratios. Longer wet curing reduced it, but under certain conditions, excess rubber could worsen chloride penetration.

In summary, rubber is hydrophobic, and an appropriate amount can boost chloride ion penetration resistance. But too much rubber may reduce this resistance.

#### 2.2.2. Freeze–Thaw Resistance

The freeze–thaw cycle refers to the process by which water freezes upon cooling below its freezing point and subsequently melts as the temperature increases. Current research suggests that RC exhibits superior freeze–thaw resistance compared to conventional concrete.

**Figure 6:**
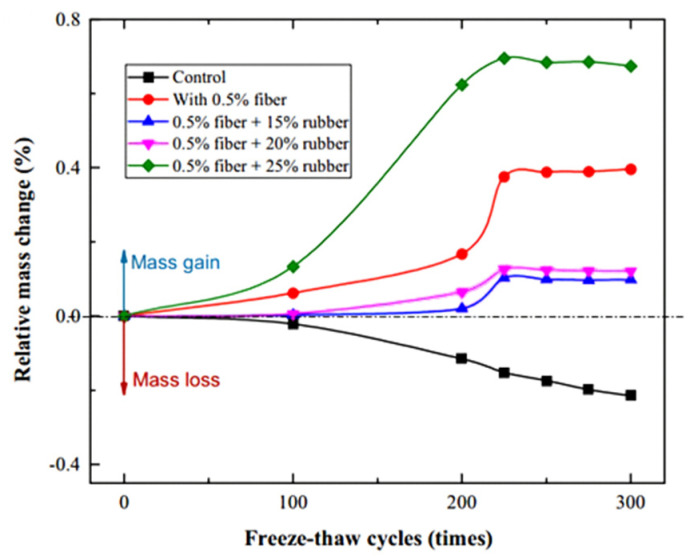
Relative mass variation of different samples [58].

Wang et al. [22] investigated the average durability coefficient of various specimens and concluded that the freeze–thaw resistance of concrete was optimized when the rubber content was 15%. However, as the rubber content increased to 25%, a decline in freeze–thaw resistance was observed, suggesting that the incorporation of an optimal amount of rubber can enhance the freeze–thaw resistance of concrete. Wang et al. [58] reached a similar conclusion in their investigation. The specimen containing 15% rubber exhibited the minimal mass change during the freeze–thaw cycle and demonstrated optimal freeze–thaw resistance (as shown in Figure 6). This investigation revealed that the incorporation of an optimal rubber content contributed to the stability of concrete quality under freeze–thaw cycles. Grinys et al. [59] demonstrated that the incorporation of rubber aggregate in concrete mitigated volume expansion and contraction under freeze–thaw conditions, with fine rubber particles having a particularly beneficial effect on freeze–thaw resistance. After 200 freeze–thaw cycles, the compressive strength of concrete specimens incorporating fine rubber increased to 49.4 MPa. Gesoğlu et al. [10] suggested that the quantity of rubber and particle size significantly influence the freeze–thaw resistance of permeable concrete. Fine rubber particles possess more micropores than their coarse counterparts. These micropores effectively alleviate concrete expansion during freezing, thereby enhancing freeze–thaw resistance. Thomas [60] also observed that concrete incorporating fine rubber particles outperformed that with coarse particles in freeze–thaw tests.

In general, both the size of rubber particles and the optimal rubber content substantially enhance the freeze–thaw resistance of concrete, with fine-grained rubber demonstrating a particularly pronounced positive effect.

#### 2.2.3. Wear Resistance

Wear resistance refers to a material’s ability to withstand wear, typically quantified by the depth of wear exhibited by concrete under standardized testing conditions. The current study posits that the incorporation of rubber can enhance the wear resistance of concrete.

**Figure 7:**
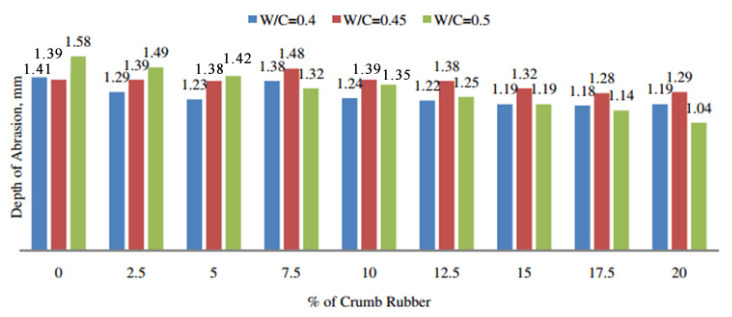
Water–cement ratio and rubber percentage RC wear resistance [14].

Thomas et al. [60] performed wear tests to evaluate the wear resistance of RC incorporating varying rubber contents. As illustrated in Figure 7, RC exhibits superior wear resistance compared to the control mix. Additionally, an increase in rubber content corresponds to a proportional enhancement in the wear resistance of RC. Kang et al. [61] prepared three mixes with varying rubber contents to investigate their effects on wear resistance. The test results indicate that as rubber content increases, the wear resistance strength systematically improves. The addition of more rubber particles results in a corresponding increase in wear resistance strength. The findings of Gesoğlu et al. [10] demonstrate that, compared to the control concrete, the wear rate decreased by 39% and 58% at 10% and 20% replacement levels, respectively. These results highlight that the incorporation of rubber positively influences the wear resistance of concrete. Gupta et al. [56] prepared concrete specimens with varying water–cement ratios (w/b) and distinct types of rubber particles and performed wear resistance tests. The results indicate that replacing fine aggregate with rubber powder and increasing the w/b ratio diminishes its wear resistance.

Based on the aforementioned research findings, it is evident that the incorporation of rubber significantly enhances the wear resistance of concrete. The size of rubber particles influences its impact on concrete, with larger particle sizes exerting a beneficial effect; however, excessively small particle sizes (e.g., rubber powder) may impair wear resistance.

### 2.3. Modification Methods of RC

#### 2.3.1. Rubber Granule Pretreatment

Untreated rubber possesses intrinsically low strength and inadequate bonding properties, prompting extensive research into various pretreatment methods to enhance its performance in RC.

**Figure 8:**
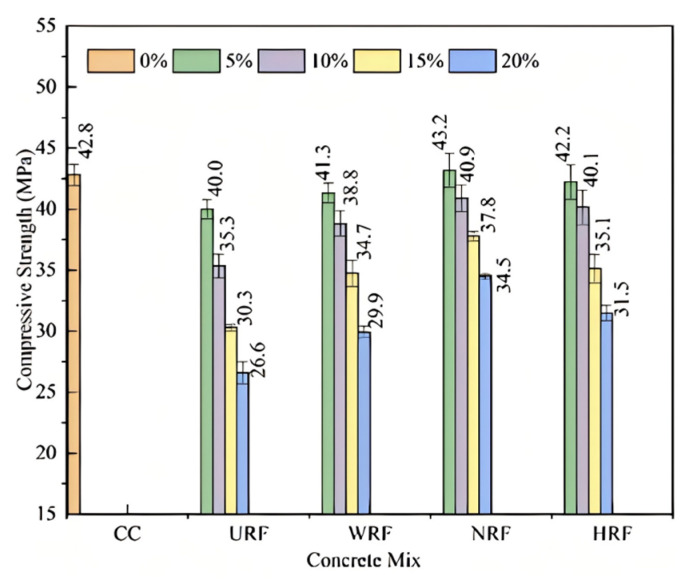
Comparison of compressive strength of concrete mixtures with different pretreatment methods [20].

Shahzad et al. [62] studied the impact of sodium hydroxide (NaOH) pretreatment on rubber particles in RC. The experimental results showed that NaOH pretreatment significantly improved the mechanical properties of high-rubber-content concrete compared to untreated concrete. There is a notable approximately 20% strength increase, especially after a 24-h pretreatment. Agrawal et al. [20] also explored the effects of NaOH pretreatment on RC. They found that after pretreatment, the compressive strength and elastic modulus of concrete with 5% rubber replacement were nearly the same as the control sample, while the splitting tensile strength and flexural strength increased by about 15% (specific data in Figure 8). Najim et al. [63] compared several pretreatment methods. They observed that NaOH pretreatment led to a small 3.1% increase in compressive strength, while cement and mortar pre-coating had much greater effects, increasing compressive strength by 15.6% and 40.6%, respectively. Li et al. [64] found that adding NaOH-treated rubber particles and cement pre-coating to concrete reduced crack width, length, and number, resulting in a more uniform crack distribution. Segre et al. [65] pretreated rubber particles with a saturated NaOH solution before adding them to concrete. Their results indicated that the treated rubber particles significantly reduced the mass loss of concrete during wear testing compared to untreated samples.

Based on the aforementioned research findings, surface treatment of rubber particles using NaOH pretreatment, along with cement and mortar coating, significantly enhances the mechanical properties of RC.

#### 2.3.2. Adding Other Ingredients

The incorporation of rubber into concrete has been shown to enhance its toughness; however, it simultaneously leads to a notable reduction in its mechanical properties, including compressive and tensile strengths. Consequently, numerous studies have explored the addition of alternative materials, such as slag, kaolin, fly ash, and silica fume, to enhance the performance of RC, specifically aiming to mitigate the negative impact of rubber on the concrete’s mechanical properties.

Raffoul et al. [66] employed silica fume and fly ash as partial replacements for cement, resulting in an optimized mixture that markedly enhanced the mixing performance of RC. The incorporation of silica fume and fly ash effectively mitigated the separation of the optimized mixture and enhanced its cohesion. Ismail et al. [67] investigated the impact of various supplementary binders on the properties of RC. The results demonstrated that with an increase in rubber content from 20% to 40%, kaolin significantly enhanced the compressive strength, splitting tensile strength, flexural strength, and elastic modulus of the self-consolidating RC mixture by an average of 49.2%, 17%, 14.6%, and 24.9%, thus mitigating the degradation of the mechanical properties of RC. Subsequent studies by Abdel Aleem et al. [68] compared the effects of silica fume, kaolin, fly ash, and slag on self-consolidating RC and found that both silica fume and kaolin enhanced the stability and mechanical properties of RC; however, they also reduced the fluidity of the mixture. Therefore, silica fume exhibits a more pronounced effect on improving fluidity compared to kaolin.

Based on the aforementioned studies, the incorporation of silica fume, metakaolin, fly ash, and slag has been shown to enhance the performance of RC across multiple parameters. The selection and incorporation of specific ingredients should be determined based on the precise engineering demands to optimize performance outcomes.

### 2.4. Summary of RC Performance Characteristics

(1) The compressive, tensile, and flexural strengths of RC are lower than those of conventional concrete, with the reduction becoming more pronounced as the rubber content increases, reaching a maximum decrease of 84%. However, replacing coarse rubber with fine rubber has been shown to mitigate the negative impact of rubber aggregates on RC’s mechanical performance. The maximum fracture energy of RC can reach 300.6 Nm/m^2^—twice that of ordinary concrete—demonstrating its exceptional toughness. In impact tests, the maximum destructive energy of RC reaches 557 J, significantly exceeding that of ordinary concrete (127 J). These results highlight RC’s superior toughness and impact resistance, making it a suitable building material for earthquake-prone regions and modern electrified railway infrastructure.
(2) RC exhibits enhanced durability compared to ordinary concrete. Its chloride ion diffusion coefficient is reduced by 24.5%, indicating improved resistance to chloride penetration. After 200 freeze–thaw cycles, concrete specimens modified with fine rubber showed a 5.6% increase in compressive strength. Additionally, at a rubber content of 60%, the carbonation depth of RC was reduced by approximately 25%. These findings demonstrate RC’s superior resistance to chloride ion ingress, freeze–thaw cycles, and carbonation, making it a viable construction material for extremely cold regions and offshore mining environments.
(3) To address RC’s lower mechanical properties, various modification techniques are employed to enhance performance. Rubber particle pretreatment methods, such as NaOH treatment and cement or mortar coating, are particularly effective. Studies have shown that NaOH pretreatment significantly improves the mechanical properties of high-rubber-content concrete under specific conditions. Similarly, cement and mortar coatings can increase compressive strength by up to 40.6%. The incorporation of supplementary materials, including silica fume, kaolin, fly ash, and slag, further enhances RC’s mechanical properties. For instance, the addition of kaolin has been found to increase compressive strength by 49.2%, tensile strength by 17%, and flexural strength by 14.6% on average.

## 3. PFRC

### 3.1. Polypropylene Fiber-Reinforced Concrete

#### 3.1.1. Micromorphology Analysis

**Figure 9:**
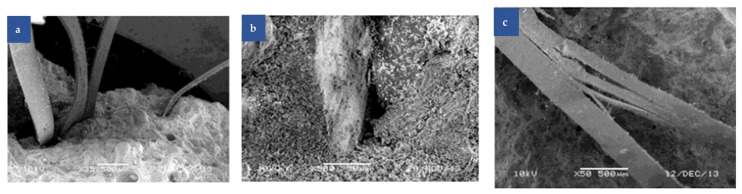
(**a**) Fiber anchored; (**b**) Pulling out fiber; (**c**) Splitting fiber [69].

Ahmad et al. [69] used SEM for a comprehensive study of fiber morphology and distribution. Figure 9a shows the SEM micrograph of the sample’s fracture surface after mechanical testing. Fibers emerge from the cement matrix in different orientations and lengths, dispersing uniformly throughout the sample. The protruding fiber ends are firmly anchored in the mortar matrix, making manual removal impossible. As seen in Figure 9b, voids are left in the matrix after fiber extraction. At the same time, the fiber network structure seems partially disrupted in the concrete matrix. Figure 9c shows fibers further fragmenting into smaller fibrils, increasing the fiber specific surface area and significantly improving adhesion properties. The interaction mechanism between fiber and matrix, including interfacial adhesion and mechanical anchoring, was clarified. Qin et al. [70] drew similar conclusions by analyzing SEM micrographs of PC and polypropylene fiber—reinforced concrete (PPFRC). In PC, the main fracture occurs at the cement–gravel interface, creating many pits in the cement, with some gravel displaced under compressive load. Clearly, the weakest part of PC is the interfacial transition zone between cement and gravel. In contrast, in PPFRC, the fibers, like steel bars, firmly anchor the cement and gravel at the microscopic level. This phenomenon greatly reduces crack propagation in the cement matrix, further indicating that fiber bonding in the concrete matrix is closely related to mechanical anchoring.

#### 3.1.2. Compressive Properties

The incorporation of polymer fibers has a complex impact on the compressive strength of concrete. On the one hand, fibers increase air voids within the mixture by altering the internal structure and facilitating air retention. On the other hand, they effectively mitigate crack propagation by acting as a bridging mechanism that inhibits further cracking. The interplay of these factors makes the overall effect on compressive strength uncertain, potentially leading to either an increase or a decrease [71].

**Figure 10:**
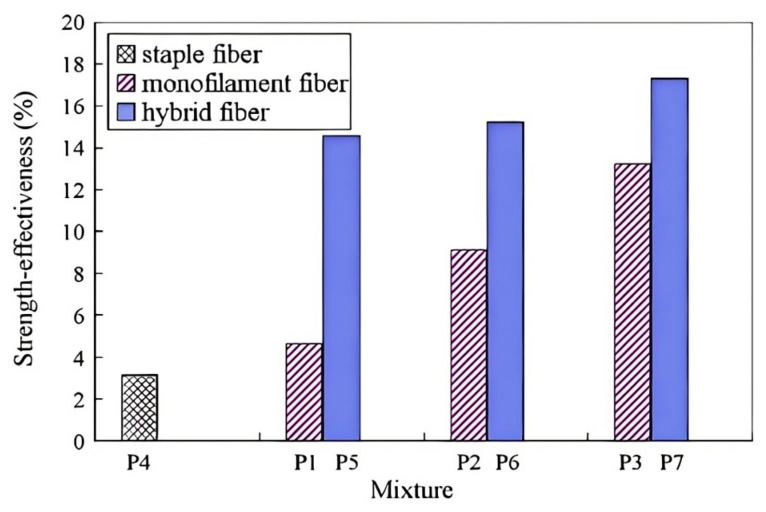
Effect of polypropylene hybrid fibers on the compressive strength of concrete [23].

Al-Katib et al. [72] conducted a comparative analysis of concrete samples with varying amounts of polypropylene fiber (PPF) and observed a progressive increase in compressive strength as PPF content increased. However, beyond a 3.5% fiber content, the rate of strength improvement plateaued, with the maximum enhancement ranging between 4% and 24%. Similarly, Hsie et al. [23] demonstrated that incorporating PPF into concrete significantly enhanced compressive strength. Their experimental results showed a positive correlation between fiber content and compressive strength, with increases of 4.65%, 9.12%, and 13.24% for fiber dosages of 3 kg/m^3^, 6 kg/m^3^, and 9 kg/m^3^, respectively (Figure 10). Abousnina et al. [25] further corroborated these findings, reporting that compressive strength progressively increased with the fiber content. Specifically, fiber dosages of 4 kg/m^3^ and 6 kg/m^3^ resulted in strength enhancements of 9.6% and 19.4%, respectively, compared to standard concrete.

However, several studies have suggested that increasing the fiber content may reduce compressive strength, indicating that PPF can negatively affect concrete’s mechanical properties under certain conditions. Islam et al. [28] reported a slight decline in compressive strength with increasing fiber content, with the most significant reduction (approximately 10%) observed at a fiber content of 0.3%. Sarikaya et al. [73] also found that a higher PPF content led to a progressive decrease in compressive strength. Their experimental data showed that the maximum compressive strength of fiber-reinforced concrete was 45.88 MPa, compared to 49.76 MPa for fiber-free concrete. The researchers attributed this decline to potential changes in matrix density, which may have limited the expected reinforcement effect.

Overall, while lower PPF dosages have minimal impact on compressive strength, an optimal dosage can significantly enhance concrete’s mechanical properties. However, the effects vary considerably depending on fiber content, highlighting the need for further research to determine the ideal dosage and its influence on concrete performance.

#### 3.1.3. Splitting Tensile Strength

Existing research indicates that an appropriate amount of polypropylene fiber (PPF) can significantly enhance the tensile strength of concrete.

**Figure 11:**
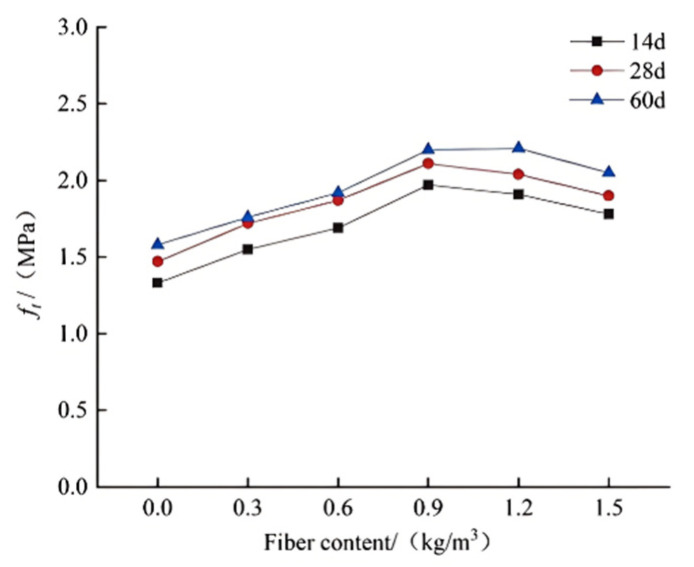
Effect of PPF content on tensile strength [34].

Zhang et al. [34] investigated the effect of PPF on concrete’s tensile strength and found that its incorporation notably improved tensile performance, imparting plastic characteristics upon failure. As fiber content increases, tensile strength initially rises before declining (Figure 11), suggesting that while moderate fiber addition strengthens the material, excessive amounts may have a detrimental effect. Bagherzadeh et al. [74] examined PPF-reinforced concrete with varying fiber ratios and aspect ratios. Their results showed that compared to the control specimen (P0), specimens with high aspect ratio fibers (P2) and high volume fraction fibers (P3) exhibited tensile strength increases of 7.36% and 15.79%, respectively (Table 4), underscoring the beneficial impact of PPF on tensile properties. Similarly, Saidan et al. [75] analyzed the influence of fiber diameter and found that concrete reinforced with large-diameter PPF achieved a tensile strength of 5.63 MPa, representing a 2.21 MPa increase over conventional concrete, whereas small-diameter PPF yielded a tensile strength of 3.70 MPa. This suggests that fiber diameter plays a crucial role in tensile strength enhancement, with larger diameters proving more effective. Yang et al. [33] explored the effects of the fiber volume fraction and loading rate on the dynamic tensile strength of PPF-reinforced cementitious composites, revealing that an optimal fiber content (1.5–2%) significantly enhanced dynamic tensile performance. Blazy et al. [24] further emphasized that an appropriate PPF dosage can substantially improve tensile strength, reinforce mechanical properties under tensile stress, and reduce the risk of failure due to tensile damage.

In summary, PPF has a positive influence on concrete’s tensile properties by mitigating crack propagation and enhancing structural integrity. However, factors such as fiber content, length, and diameter require careful optimization to maximize performance.

#### 3.1.4. Crack Resistance

Adding PPF to the cement matrix can significantly lower concrete’s free shrinkage, thus improving its crack resistance.

**Figure 12:**
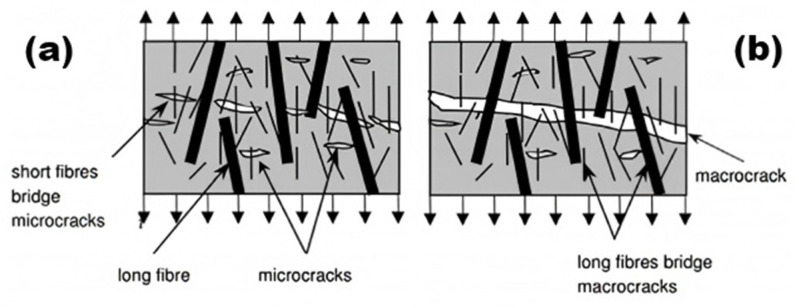
(**a**) Effect of shorter fibers on bridging microcracks and improving tensile strength; (**b**) Effect of longer fibers on bridging macrocracks and improving ductility [24].

Blazy et al. [24] showed that freshly placed concrete had low initial strength and elastic modulus. Stresses from plastic shrinkage can easily cause cracks. A large amount of evenly distributed PPF can notably decrease crack width and even bridge cracks to stop further spread (Figure 12). The results indicated that fiber-free concrete had a large crack area, but with 0.05% and 0.1% PPF added, the crack area was greatly reduced, with crack reduction factors of 43% and 94%, respectively. Zhu et al. [27] also found that PPF incorporation significantly boosted concrete’s crack resistance. Especially in the early stages of concrete hardening, PPF effectively prevents the formation of early plastic shrinkage cracks. The three-dimensional network formed by fibers in the concrete further enhances its crack resistance. Wang et al. [22] demonstrated that PPF-reinforced concrete had far better crack resistance than conventional concrete, about 15 times greater (ranging from 0.133 N/mm to 2.03 N/mm).

Existing research indicates that the incorporation of PPF not only significantly mitigates early plastic shrinkage cracks in concrete but also improves its overall crack resistance through the three-dimensional network structure formed within the matrix.

#### 3.1.5. Toughness

Toughness refers to a material’s ability to absorb energy from the onset of environmental loading to the point of failure, thereby reflecting its capacity to deform under stress.

**Figure 13:**
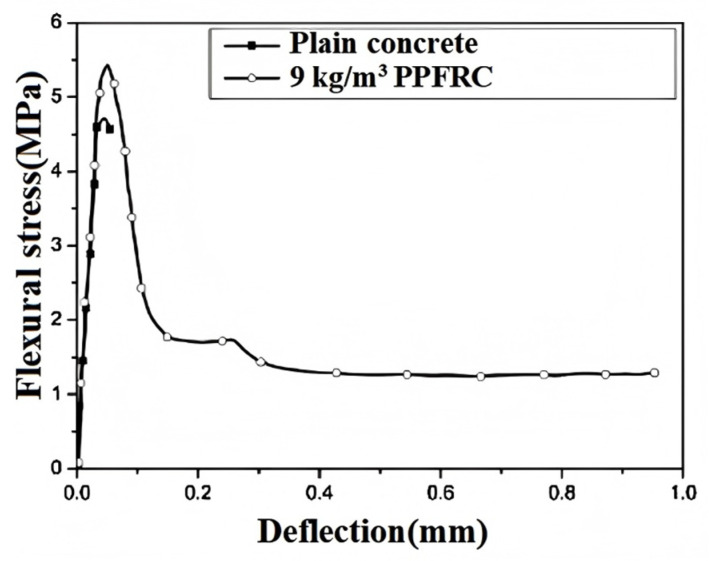
Flexural stress-deflection curves of PPFRC and plain concrete [24].

Blazy et al. [24] showed that adding PPF greatly improved concrete’s toughness. Conventional concrete has a toughness index of 0, while that of concrete with 0.5% and 1.1% PPF reaches 22% and 38%, respectively. After reaching the peak and the first crack, PPF-reinforced concrete (PPFRC) does not fail brittlely but keeps transferring stress (Figure 13), meaning that PPF allows concrete to absorb more energy during failure, enhancing toughness. Zhang et al. [26] used the energy method to measure PPFRC’s toughness and found that it was strongly affected by the strain rate and fiber content. As the strain rate rises, toughness increases, with an optimal fiber content for maximum gain. Zhu et al. [27] also observed that PPFRC’s peak and ultimate toughness increased with the strain rate. In their experiments, higher strain rates mean greater impact loads, shorter crack-propagation times, more new cracks, and thus more energy absorption, raising toughness. The results showed that PPF significantly boosted concrete’s toughness.

These studies demonstrate that the incorporation of PPF into concrete positively influences its toughness through various mechanisms. It can interact with other constituents in the concrete matrix, enhancing its ability to resist stress and deformation, and thereby improving the overall toughness.

#### 3.1.6. Durability Performance

Suiffi et al. [76] showed that adding PPF significantly improves concrete’s durability, especially its fire resistance. Tests revealed that incorporating PPF into the cement matrix boosts fire resistance, with the best performance at a 0.50% PPF volume fraction. Liu et al. [29] tested chloride ion permeability resistance to assess PPF’s effect on durability. They found that as the fiber volume fraction rose, the chloride ion migration depth in concrete gradually dropped, meaning that PPF effectively enhanced chloride ion penetration resistance and durability. Li et al. [77] analyzed crack geometry and permeability. While increased crack tortuosity lengthens the actual crack length, more pathways for harmful substances are created. But increased crack surface roughness blocks their penetration, improving durability. Water permeability tests showed that PPF reduces concrete’s water permeability, limiting water and harmful ion ingress. Wang et al. [78] demonstrated that the fiber volume content significantly impacted PPFRC’s durability. During dry–wet cycles, an optimal 0.1% fiber content reduces water absorption and chloride ion content while increasing density. During freeze–thaw cycles, fibers enhance freeze–thaw resistance; for example, the 0.5% fiber specimen had the lowest spalling rate and smallest relative dynamic elastic modulus decrease. However, too much fiber raises the chloride ion diffusion coefficient.

In conclusion, adding PPF greatly enhances concrete durability, specifically in fire, permeability, chloride ion penetration, freeze–thaw, sulfate, and acid corrosion resistance.

### 3.2. Polyvinyl Alcohol Fiber Reinforced Concrete

#### 3.2.1. Micromorphology Analysis

**Figure 14:**
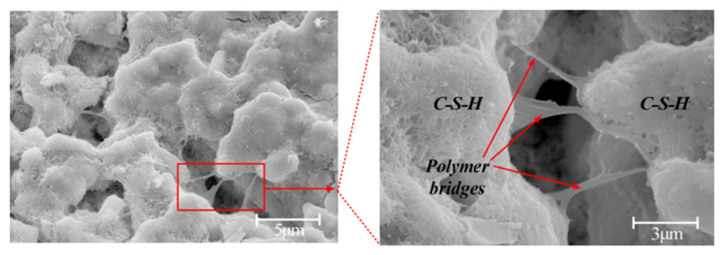
Polymer film stretched in open space in 0.6% PVA modified mortar at 28 days [79].

Fan et al. [79] observed through SEM that polyvinyl alcohol (PVA) formed a uniformly distributed film network within the cement matrix. These network structures not only enhance the connectivity between cement hydration products but also play a critical “bridging” role at microcracks, thereby helping to inhibit both the initiation and propagation of cracks, as depicted in Figure 14. Tan et al. [80] investigated the internal structure of polyvinyl alcohol fiber-reinforced concrete (PVAFRC). Compared to ordinary concrete, the interface transition zone between polyvinyl alcohol fibers (PVAF) and the cement paste was significantly enlarged. The reverse stress field formed at the fiber tip effectively prevented crack initiation. The bridging effect of the fiber enabled both the fiber and the surrounding cementitious material to form a composite force system. The formation of this force system substantially enhanced the load-bearing capacity of the concrete. Furthermore, Cheng et al. [81] emphasized that PVAF plays a crucial bridging role in cement-based composite materials, effectively limiting crack propagation and enhancing both the toughness and ductility of the material.

Due to its high strength and modulus characteristics, PVAF is effectively integrated with the cement-based composite matrix to form a three-dimensional supportive structure. When stress is applied, the fiber bears a portion of the tensile stress, thereby delaying crack propagation.

#### 3.2.2. Compressive Properties

**Figure 15:**
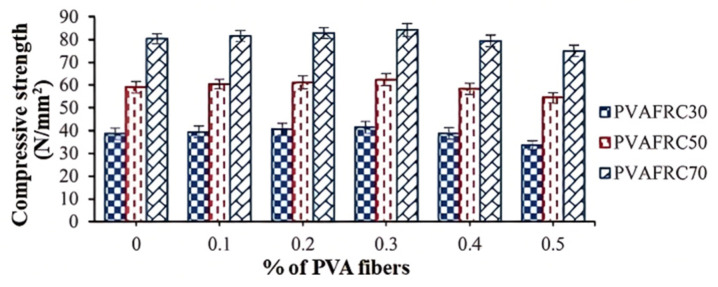
Effect of PVA fiber addition on properties of three grades of PVAFRC. Compressive toughness index [83].

Research has shown that adding PVAF has a limited impact on enhancing concrete’s compressive strength. Low levels of polymer fibers can boost this strength, but too much fiber may cause it to drop. Xiao et al. [82] tested the compressive performance of PVAF-reinforced concrete (PVAFRC) with different fiber contents. When the PVAF content was 1.2%, the compressive strength increased by only 1.7 MPa compared to non-PVAF concrete, suggesting that as PVAF content rose, the improvement in compressive performance was restricted. Sagar et al. [83] found that adding PVAF to concrete increased strength by 5–7% when the fiber content was 0.3%. But increasing it to 0.4% or 0.5% decreased the compressive strength (Figure 15). This drop may be due to excessive fiber reducing concrete vibration and compactness, increasing porosity. Noushini et al. [84] obtained similar results. When 6-mm long PVAF was added at 0.25%, the concrete’s compressive strength reached its maximum, about 10 MPa higher than without PVAF. Further increasing the dosage decreased the strength. Fan et al. [79] also showed that as PVA content increased, concrete strength first rose then fell. At a 2% PVA dosage and 28-day curing, compressive strength peaked; any more led to a decrease, even below that of fiber-free concrete.

In conclusion, the PVAF dosage greatly affects concrete’s compressive strength. The optimal dosage is usually between 0.25% and 0.3%. Going beyond this range may degrade concrete performance.

#### 3.2.3. Splitting Tensile Strength

The addition of an appropriate amount of PVAF to concrete can substantially enhance its tensile strength. According to the studies by Shi [85], Wang [86], and Lin [87], when the PVAF content ranges from 0% to 2.5%, the tensile strength of PVAFRC initially increases and then decreases, with the optimal effect occurring when the PVAF content is between 1.5% and 2%. A study by Yew et al. [88] revealed that when the fiber content increased from 0% to 0.5%, the 28-day splitting tensile strength rose from 2.88 MPa to 3.74 MPa. The results indicate that the splitting tensile strength increases with the rise in PVAF volume fraction. A study by Noushini et al. [84] demonstrated that when the fiber length was 6 mm and the volume fraction was 0.25%, the tensile strength achieved optimal performance, with the splitting tensile strength increasing by an average of 30%. The experimental results from Shi et al. [85] also indicated that the incorporation of 1.5% PVAF can substantially enhance the tensile properties of concrete while improving its ductility. Collectively, these experimental findings demonstrate that the tensile strength of concrete is markedly improved when an optimal amount of PVAF is incorporated.

#### 3.2.4. Flexural Strength

Adding PVAF greatly improves the flexural properties of concrete. Research by Shi [85], Wang [86], and Lin [87] showed that the PVAF dosage significantly affected the flexural strength of concrete. When the PVAF content varies from 0% to 2.5%, the flexural strength of PVAFRC first increases and then decreases with rising fiber content. The optimal PVAF content lies between 1.5% and 2%. In this range, PVAF is distributed evenly in the matrix, maintaining the concrete’s isotropic mechanical properties and maximizing the fiber-bridging effect, thus enhancing the flexural properties significantly. Harun’s research [89] also revealed that as the PVA content increased, the flexural strength improved. When the fiber content reached 2%, the concrete’s tensile strength was much higher than that of plain concrete.

According to Noushini et al. [84], PVAFRC showed a remarkable increase in flexural strength. At 28 days, the flexural strength was 11–21.5% higher than that of the control group. The most significant improvement in flexural strength occurred when the PVAF content was 0.25%.

Generally, within the optimal dosage range, PVAF fibers enhance the flexural strength of concrete by providing additional bridging effects and promoting multiple crack propagation mechanisms. However, exceeding the optimal fiber content may result in an uneven fiber distribution and improper orientation, leading to detrimental effects. Therefore, in practical applications, it is necessary to adjust the fiber content proportion in accordance with specific requirements to optimize the flexural properties of concrete.

#### 3.2.5. Durability Performance

Adding PVAF greatly improves concrete’s frost resistance. This is mainly because PVAF is evenly distributed, effectively countering the tensile stress from frost heave. It thus reduces crack propagation and withstands the expansion pressures during freeze–thaw cycles.

Zhao et al. [90] found that the ideal PVAF content was 0.15%. After freeze–thaw cycles, the compressive strength reduction rate of PVAFRC was only 35%. Tan et al. [80] further showed that as PVAF content rose, the mass loss rate of concrete after freeze–thaw cycles gradually dropped, aggregate spalling lessened, and the reduction in relative dynamic elastic modulus became less significant.

**Figure 16:**
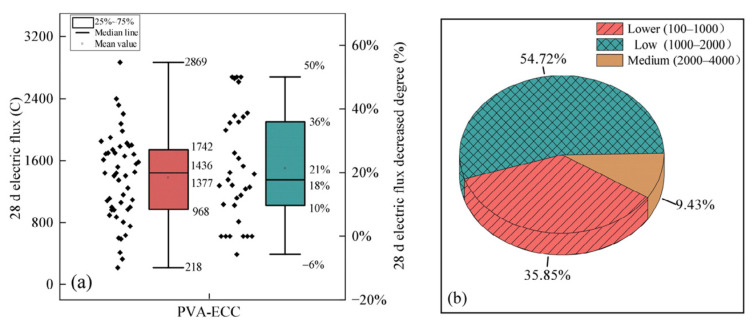
PVAFRC resistance to chloride ion penetration: (**a**) PVAFRC electric flux; (**b**) evaluation distribution of chloride ion permeability of PVAFRC [81].

Regarding chloride ion permeability resistance, research by Zhang [91] and Cheng [81] indicated that PVAF-incorporated concrete resisted chloride ion penetration better. As seen in Figure 16, at 28 days, the electrical flux of PVAFRC is 10–36% lower than that of ordinary concrete. Electrical flux data also show that PVAFRC has good chloride ion permeability performance, with 54.72% of the data in the low-permeability range and only 9.43% in the medium-permeability range. The even spread of PVAF in the matrix forms a support system that strengthens, stabilizes, and anchors, effectively bridging micro-cracks and controlling crack growth. This curbs chloride ion penetration and migration, enhancing the concrete’s permeability resistance. Noushini et al. [84] noted that low content PVAF significantly boosted concrete’s crack and erosion resistance, especially at lower contents. But when PVAF content exceeded 0.5%, the improvement in concrete durability started to wane, and increased porosity may affect permeability resistance.

Consequently, the judicious incorporation of PVAF can markedly enhance the frost resistance, chloride ion permeability resistance, and crack resistance of concrete. The optimal dosage range lies between 0.1% and 0.3%, with any content beyond this range potentially leading to a decline in performance.

### 3.3. Polymer Hybrid Fiber Reinforced Concrete

#### 3.3.1. Compressive Properties

Research has shown that incorporating polymer hybrid fibers can significantly boost concrete’s compressive properties more than single polymer fibers.

**Figure 17:**
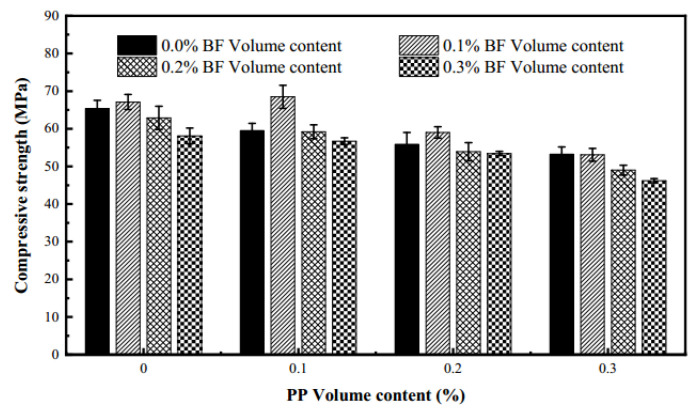
Compressive strength of basalt fiber-reinforced concrete [96].

Singh et al. [92] studied the effect of combining steel and polypropylene fibers (PPF) on concrete’s compressive strength. Steel–polypropylene hybrid fiber-reinforced concrete had better strength than single fiber-reinforced concrete. At higher fiber volume fractions, it showed enhanced synergistic effects, meaning an optimal amount could lead to greater strength improvements. Afroughsabet et al. [93] found that adding hybrid fibers (steel and polypropylene) significantly increased concrete’s compressive strength, which rose as the fiber volume fraction increased. The strength peaked when the ratio of steel to polypropylene fibers was 0.85% and 0.15%. Guo et al. [94] observed that as the steel fiber content increased, concrete’s compressive strength improved. But polypropylene fiber content first enhanced then decreased the strength. Steel fibers strengthen the matrix, while polypropylene fibers may increase porosity, reducing strength. Shi et al. [95] showed that adding basalt and polypropylene fibers together significantly increased compressive strength, with basalt fibers having a more prominent reinforcing effect. Wu et al. [96] also found that combining polypropylene and basalt fibers improved concrete’s compressive strength (Figure 17).

In conclusion, polymer hybrid fibers benefit concrete’s compressive strength within an optimal dosage range. Excessive fiber can harm mechanical properties. Different fiber combinations have varying impacts, so optimizing the combination and proportion is crucial for better concrete performance.

#### 3.3.2. Splitting Tensile Strength

**Figure 18:**
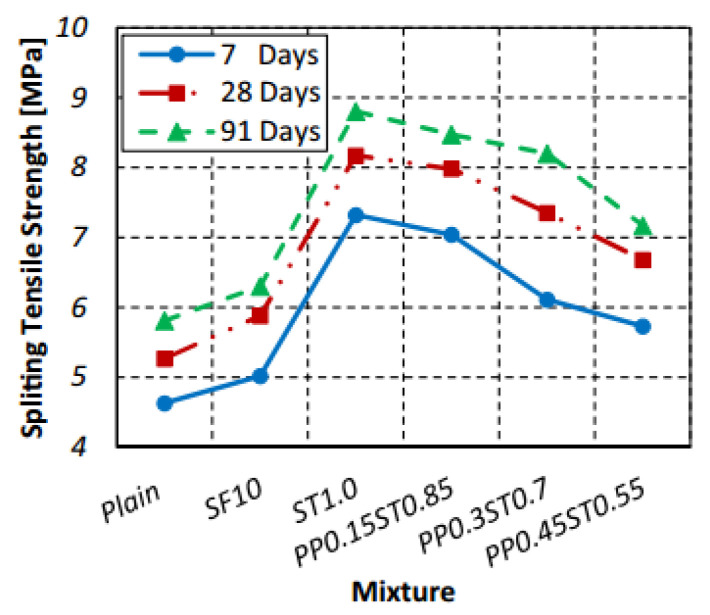
Splitting tensile strength of concrete of hybrid fiber-reinforced specimens [93].

The addition of polymer hybrid fibers can markedly boost the tensile strength of concrete. Niu et al. [97] found that in basalt–polypropylene hybrid fiber-reinforced concrete, the synergistic effect between basalt fiber and PPF improved the concrete’s microstructure and strengthened the bond between fibers and the concrete matrix, enhancing its tensile properties. Kanagavel et al. [98] experimentally studied the mechanical properties of cement concrete with hybrid fibers. The results showed that within a certain dosage range, polymer hybrid fibers (steel fibers and PPF) greatly increased the splitting tensile strength of concrete. Specifically, it can be up to 94% higher than that of the control concrete. Afroughsabet et al. [93] examined the mechanical properties of steel–polypropylene hybrid fiber-reinforced concrete via a splitting tensile strength test. The study indicated that the splitting tensile strength increased by 23–52%. The optimal mixture, with 0.15% PPF and 0.85% steel fiber, most effectively enhanced the concrete’s tensile strength (Figure 18). The tensile strength of polymer hybrid fiber-reinforced concrete is affected by multiple factors. Adding the right amount of fiber can notably boost the concrete’s tensile properties. Fiber type and quantity are key determinants, and choosing different hybrid fiber combinations is of great importance. A well-designed combination can have a synergistic effect, significantly increasing the tensile strength. Overall, by carefully selecting and optimizing polymer hybrid fibers, the tensile strength of concrete can be greatly enhanced, improving its performance in engineering applications.

#### 3.3.3. Toughness

**Figure 19:**
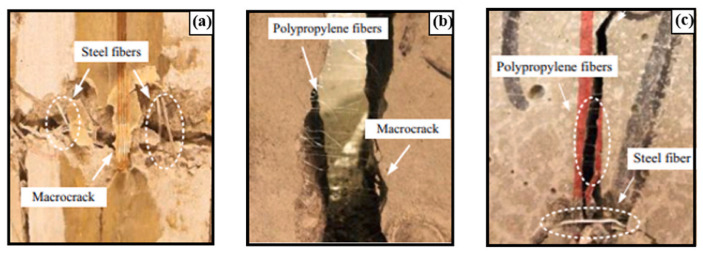
Fiber-bridging effect image: (**a**) Cracked cross section of steel fiber-reinforced concrete; (**b**) Cracked section of concrete containing PPF; (**c**) Hybrid fiber crack cross-section [99].

The addition of polymer hybrid fibers can greatly enhance the toughness of concrete. Liang et al. [99] showed through experiments that concrete’s flexural strength is closely linked to its toughness. Fibers not only boost flexural strength but also significantly increase toughness. In polypropylene–basalt fiber-reinforced concrete experiments, fiber addition improved the material’s flexural strength. Under bending load, fibers effectively reduced crack propagation, showed enhanced ductility, and thus improved the material’s toughness (Figure 19). Shi et al. [95] demonstrated that hybrid fibers create a tortuous crack propagation path. This increases resistance to crack growth and enhances the overall toughness of concrete. Compared to conventional concrete, hybrid fiber concrete has more dispersed cracking, reducing localized crack formation and promoting better energy dissipation. Test results show that hybrid fibers can significantly enhance concrete’s fracture toughness. Zeng et al. [100]’s three-point bending test indicated that fiber inclusion significantly improved the concrete’s bending performance and toughness. The load deflection curve shows that non-fiber-reinforced concrete fails brittlely, while hybrid fiber-reinforced concrete can still bear load after reaching the peak, showing improved ductility and toughness. The area under the load deflection curve is larger than that of plain concrete, indicating a greater energy absorption capacity.

Polymer hybrid fibers substantially influence the toughness of concrete. Their distinctive three-dimensional random distribution remarkably bolsters the structural characteristics of concrete, effectively impeding both the initiation and spread of cracks. Functioning as bridges and connectors within the concrete matrix, these fibers proficiently transfer and distribute stress, thus augmenting the concrete’s toughness.

#### 3.3.4. Durability Performance

Polymer hybrid fibers have a significant impact on concrete durability. By strategically combining different fiber types and sizes, concrete durability can be optimized.

Hybrid fibers greatly enhance concrete durability, especially in chloride ion penetration resistance, sulfate attack resistance, and freeze–thaw durability. Liu et al. [101] showed that combinations, like steel and polypropylene fibers, can significantly lower chloride ion migration depth and coefficient, enhancing penetration resistance. At a 1.5% fiber volume fraction, a notable reduction in migration depth occurs. Hybrid fibers also reduce pore structure and shrinkage cracks, improving overall durability. Liu et al. [102] used orthogonal experiments to study hybrid fibers’ effects on durability. Different fiber types, such as basalt and PVAF, have distinct properties and impacts on concrete. The optimal fiber content boosts chloride ion penetration resistance, while too much fiber may reduce it. Fiber bridging is crucial for this resistance. Köksal et al. [103] demonstrated that steel fiber and PPF combinations significantly affect concrete durability. Glass fiber and PPF combinations reduced chloride ion migration depth and improved impermeability at certain volume fractions. For freeze–thaw resistance, some hybrid fiber combinations enhance durability and reduce wear loss. Hybrid fibers can decrease concrete drying shrinkage but may not outperform single fibers in shrinkage and crack resistance.

In conclusion, polymer hybrid fibers substantially enhance concrete durability against chloride ion penetration, sulfate-induced degradation, and freeze–thaw cycles. The right fiber combination and dosage can refine the pore structure and improve durability, but excessive fiber can have negative effects.

### 3.4. Summary of PFRC Performance Characteristics

(1) The incorporation of polymer fibers in cementitious composites exhibits complex nonlinear relationships with compressive strength enhancement. Experimental evidence suggests an optimal dosage range achieving 5–20% strength improvement through matrix densification and microcrack restraint mechanisms. However, when the polymer fiber content surpasses a certain threshold, the compressive strength may decline due to factors such as disrupting the compactness of the concrete matrix. Therefore, it is essential to ascertain the optimal polymer fiber content.
(2) Polymer fibers can reduce crack formation and improve stress transfer under tensile forces, thereby enhancing the tensile strength of concrete by approximately 10% to 30%. However, an excessive quantity of polymer fibers can decrease tensile strength due to issues like fiber dispersion. Moreover, different types and aspect ratios of polymer fibers exhibit differential effects on tensile strength enhancement. Consequently, in engineering applications, it is important to optimize the polymer fiber parameters based on specific requirements to fully realize their reinforcing potential.
(3) Polymer fibers can significantly improve the flexural properties of concrete. Once cracks form, the bridging effect of the fibers can slow crack propagation, thereby increasing the flexural strength and enhancing toughness. Regulating the polymer fiber dosage within the range of 0.5% to 2% can increase the flexural strength of concrete by 10% to 25%. It is important to note that an excessive quantity of polymer fibers can result in fiber agglomeration and non-uniform distribution, leading to stress concentration and a reduction in flexural strength. Therefore, careful control of parameters such as polymer fiber dosage is crucial to optimizing the flexural properties of concrete.
(4) Polymer fibers can significantly enhance the durability of concrete. In terms of resistance to chloride ion penetration, polymer fibers can reduce the depth of chloride ion migration by approximately 10% to 30%. For freeze–thaw resistance, polymer fibers can withstand frost-induced tensile stress and prevent crack propagation, allowing concrete with an optimal quantity of polymer fiber content to substantially reduce the loss of compressive strength, mass, and elastic modulus after exposure to freeze–thaw cycles.

## 4. PFRRC

### 4.1. Polypropylene Fiber Reinforced-Rubber Concrete

#### 4.1.1. Compressive Properties

**Figure 20:**
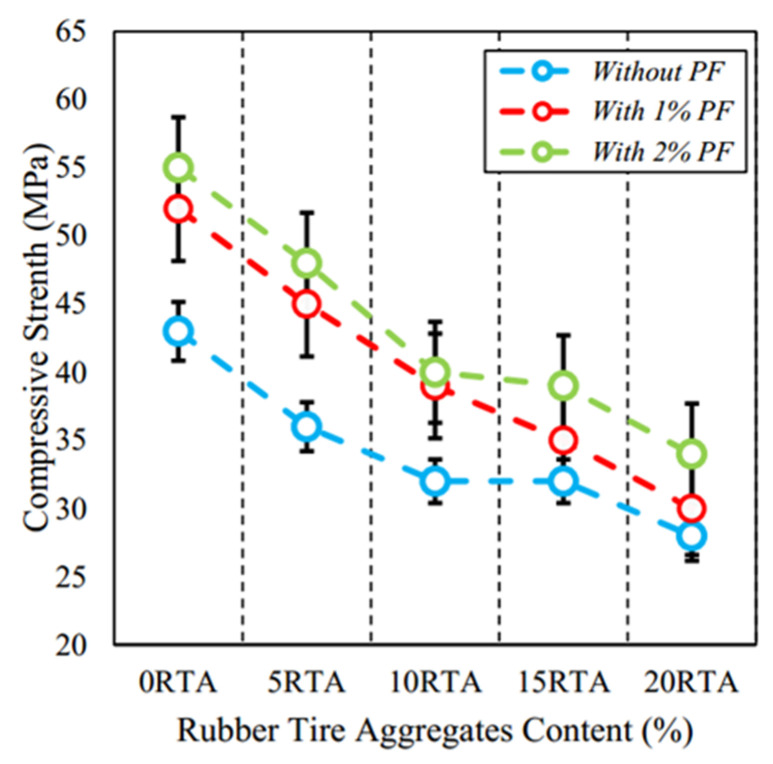
Compressive strength of concrete with different PPF and rubber tire aggregates [104].

The incorporation of PPF into RC can significantly enhance its compressive properties. Research has shown that while rubber tire aggregate in concrete may reduce compressive strength, adding PPF notably improves it. Arash et al. [104] found that with 0%, 5%, 10%, 15%, and 20% rubber tire aggregate content, the 7-day compressive strength of RC increased by 24%, 29%, 24%, 25%, and 22% respectively after adding 2% PPF (see Figure 20), suggesting that PPF can partly restore concrete’s compressive properties. Hossain et al. [105] also indicated that PPF addition effectively boosted RC’s compressive strength. As the PPF proportion rose, the compressive strength of specimens with 10% recycled aggregate replacement improved notably with 1% and 2% fiber addition. Moreover, studies have revealed a complex effect of PPF content on compressive strength. Su et al. [106] demonstrated that as the PPF content increased from 0% to 1.8%, the compressive strength gradually grew, peaking at 1.8%, but dropped significantly when it further rose to 4.51%. Zhang et al. [107] also noted that fiber content enhanced the compressive strength within a certain range; beyond that, strength declined. Han et al. [108] further confirmed the positive effect of PPF addition on RC’s compressive strength, though excessive fiber content led to a strength decrease.

Therefore, an optimal amount of PPF can significantly enhance the compressive strength of RC, primarily by establishing a network structure within the matrix through the fibers, thereby improving crack resistance. However, an excessive quantity of PPF can result in poor dispersion within the RC and the formation of matrix defects, ultimately leading to a reduction in compressive strength.

#### 4.1.2. Splitting Tensile Strength

Incorporating rubber aggregate typically reduces concrete’s splitting tensile strength, but adding PPF can offset this. Rubber particles’ bond with cement paste is prone to sudden breakage, creating a weak interfacial transition zone. Conversely, PPF’s high tensile strength enables a strong bond between aggregate and cement paste, increasing splitting tensile strength as PPF content rises.

**Figure 21:**
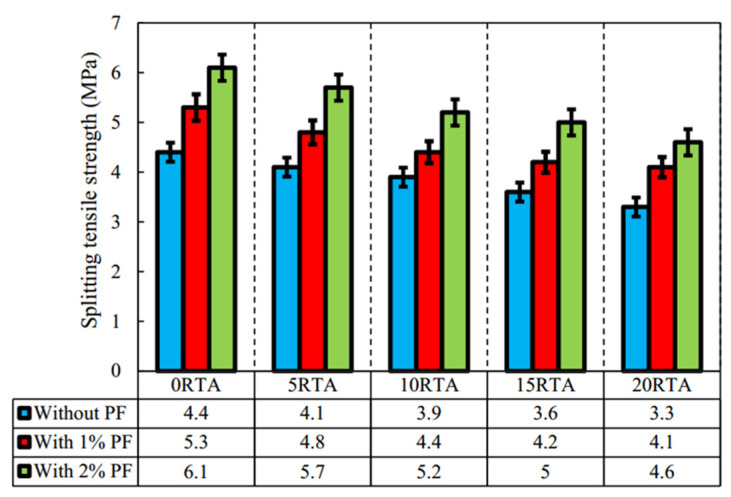
Splitting tensile strength of different fiber contents [104].

Arash et al.’s research [104] showed that using 20% rubber tire aggregate cut tensile strength by 23%. However, with 2% PPF, the splitting tensile strength increased by about 38%. PPF effectively lessened the strength loss from rubber tire aggregate. As seen in Figure 21, at different rubber tire aggregate contents (0%, 5%, 10%, 15%), adding 2% PPF raised the splitting tensile strength of RC by 28%, 18%, 13%, and 5% respectively. Zhang et al. [107] provided an empirical formula for R5 (5% rubber particle replacement) and R25 (25% rubber particle replacement) concrete, linking tensile strength and fiber content. Derived from linear fitting when the fiber content is below 0.9 kg/m^3^, in the formula, T is splitting tensile strength and d is fiber content. Tensile strength gradually increases with rising fiber content. Han et al. [108] found that different PPF contents greatly affected splitting tensile strength. As the fiber content increased, tensile strength rose significantly, with a maximum 8.14% increase at 0.2% fiber content. These results indicate that PPF can notably enhance the splitting tensile strength of RC.

#### 4.1.3. Flexural Strength

**Figure 22:**
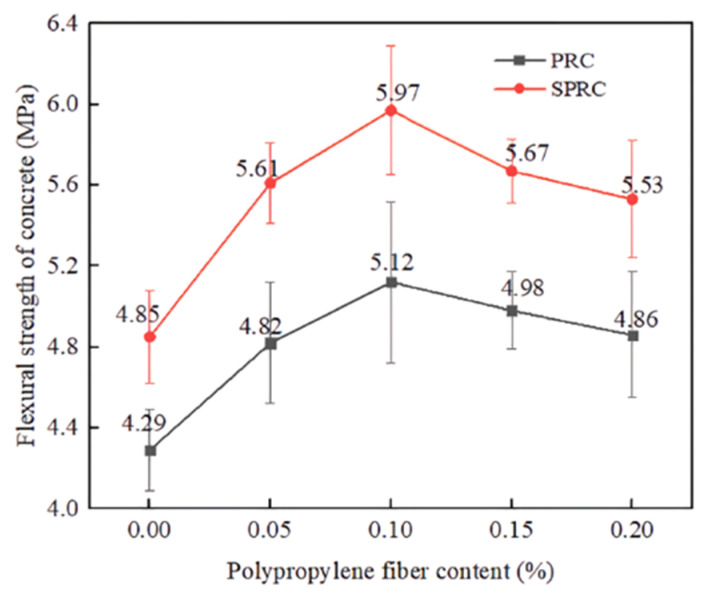
Effect of different PPF contents on flexural strength [109].

Saeid Hesami et al. [39] found that replacing 15% of sand with tire rubber reduced the flexural strength by 17%. But PPF can boost flexural strength after cracks form. Fibers act as bridges connecting cracks and withstanding loads post-crack. Arash et al. [104] showed that PPF effectively counteracted the flexural strength decrease from rubber tire aggregate. Adding 2% PPF increased flexural strength by 34%. Even at 5%, 10%, and 15% rubber tire aggregate contents, flexural strength rose by 24%, 16%, and 6% respectively (Figure 22). This is due to PPF’s bridging role, which dissipates energy and increases fracture energy. Hossain et al. [105] noted that because rubber aggregate has a lower elastic modulus than hardened cement paste, the adhesion between rubber particles and mortar is poor. So, at a constant rubber particle substitution, flexural strength increases as the PPF substitution level rises. Su et al. [109] found that the PPF content significantly impacted the flexural strength of RC. When the PPF content increased from 0.05% to 0.1%, flexural strength rose by 12.35% and 19.35% respectively. But at 0.15% and 0.2%, the increase lessened to 16.08% and 13.29%, respectively (Figure 22).

In summary, PPF can notably enhance the flexural strength of RC, especially at higher rubber aggregate replacement ratios. The right amount of PPF improves concrete’s crack-bridging ability and flexural strength. However, too much PPF may reduce its positive impact on flexural strength due to problems, like uneven dispersion and material structure flaws.

#### 4.1.4. Impact Resistance

RC has garnered significant attention due to its exceptional energy absorption and impact resistance properties. Research has indicated that the incorporation of PPF can further enhance the impact resistance of RC. Ling et al. [110] and Zhu et al. [111] reported that the impact absorption capacity of RC surpassed that of ordinary concrete. Additionally, the incorporation of PPF significantly enhanced the durability, tensile strength, impact resistance, and toughness of the cement matrix, while effectively controlling cracking and expansion. Song et al. [112] demonstrated that PPF significantly improved the impact resistance of concrete. Furthermore, the energy required for the initial cracking of concrete with added PPF was 30.5% higher than that of ordinary concrete. These findings indicate that the incorporation of PPF not only improves the impact resistance of concrete but also enhances its energy absorption capacity during the cracking process, resulting in polypropylene fiber-reinforced rubber concrete (PPFRRC), which exhibits superior performance in impact resistance. This conclusion was further corroborated by the experimental study conducted by Ankush et al. [113]. Research has demonstrated that concrete containing both rubber particles and PPF exhibits improved impact resistance and ductility.

In conclusion, the incorporation of PPF not only improves the impact resistance of RC but also significantly enhances its energy absorption capacity and ductility. Particularly when PPF and rubber particles are incorporated in optimal quantities, the impact resistance of concrete is markedly improved. However, excessive incorporation of rubber particles may result in a reduction in impact resistance. Therefore, optimizing the rubber replacement ratio and PPF dosage is crucial to enhancing the impact resistance of RC.

#### 4.1.5. Durability Performance

Most experimental findings indicate that the incorporation of PPF can enhance wear resistance by 20–60%. PPFRRC demonstrates excellent durability. According to Arash et al. [104], a 10% rubber tire aggregate content was optimal for durability, as values exceeding this threshold led to a significant decline in durability. Furthermore, the incorporation of PPF enhanced durability, particularly when 2% PPF was utilized. Moreover, the durability coefficient increased by 13%, 31%, and 37%, respectively, when 2% PPF was combined with 0%, 5%, and 10% rubber tire aggregate in concrete.

**Figure 23:**
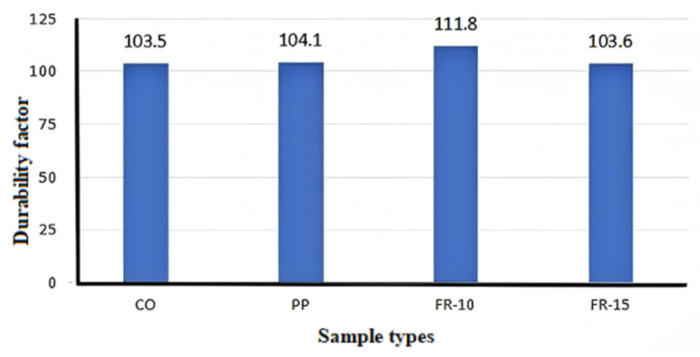
Durability factors of different types of specimens [22].

Regarding the frost resistance of PPFRRC, Wen et al. [114] conducted experiments, and their findings demonstrated that the incorporation of PPF enhanced the frost resistance of RC. These findings suggest that PPFRRC has significant potential for application in frost-resistant engineering. Wang et al. [22] demonstrated through experiments the calculated average durability factors for various specimens, as depicted in Figure 23. A higher durability factor correlates with superior freeze–thaw resistance. The durability factor of the FR-10 (PPFRRC with 10% rubber aggregate) group samples is notably high. The inclusion of low content fine rubber aggregate enhances freeze–thaw resistance, resulting in superior durability in freezing and wet conditions. These results indicate that the increased air content resulting from the introduction of rubber aggregate may contribute to enhanced freeze–thaw resistance. The improvement in freeze–thaw durability is more pronounced in PPFRRC containing 10% rubber aggregate.

Therefore, the incorporation of PPF not only improves the wear resistance and frost resistance of RC but also significantly enhances the overall durability of the concrete matrix. When an appropriate amount of rubber aggregate is incorporated, PPFRRC demonstrates excellent performance in freeze–thaw resistance and durability, offering promising prospects, particularly in projects demanding high durability and frost resistance.

### 4.2. Polyvinyl Alcohol Fiber Reinforced-Rubber Concrete

#### 4.2.1. Compressive Properties

**Figure 24:**
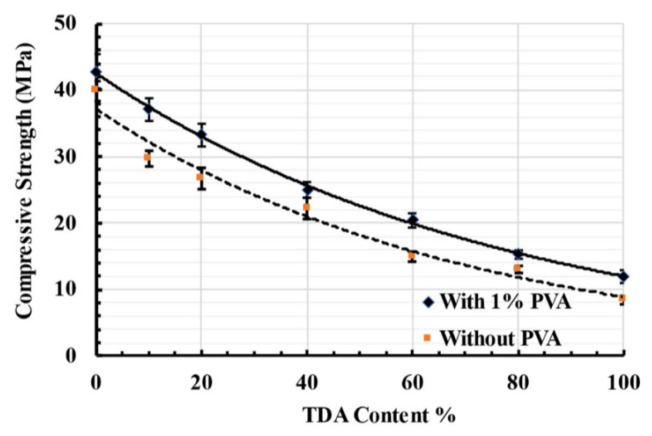
Relationship between compressive strength and TDA content and PVA percentage [115].

The experimental findings of Naggar et al. [115] showed that incorporating 1% PVAF mitigated the strength loss in RC. As shown in Figure 24, the loss of compressive strength in tire-derived aggregate (TDA) concrete was significantly reduced compared to the control group without PVAF. Specifically, the TDA20%-1%PVA specimen exhibited a compressive strength loss of only 22%, notably lower than the 33% observed in the TDA20% PVA specimen without PVAF. These results indicate that PVAF can enhance the compressive strength of RC and mitigate the adverse effects of rubber.

Similarly, Feng et al. [37] reported that at a rubber replacement ratio of 20%, incorporating 0.5% and 1.0% PVAF increased the 28-day compressive strength to 17.75 MPa and 18.01 MPa, respectively. However, at 1.5% PVAF, the strength decreased to 16.87 MPa, suggesting that an optimal PVAF dosage exists, beyond which strength enhancement diminishes. Ma et al. [116] further validated this conclusion, demonstrating that when PVAF and rubber powder were co-added, the axial compressive strength was lower than that of the benchmark group (P0R0). Moreover, increasing the fiber content led to a decrease in compressive strength, reinforcing the nonlinear relationship between PVAF dosage and RC strength.

Additionally, Liu et al. [117] examined the influence of PVAF length on RC compressive strength. For concrete containing 15% rubber, an increase in PVAF dosage initially improved compressive strength, peaking at a volume dosage of 1% before declining. When the dosage remained constant, the 9-mm PVAF length yielded the most favorable results. These findings highlight the critical role of both PVAF length and dosage in optimizing RC’s mechanical properties.

#### 4.2.2. Splitting Tensile Strength

**Figure 25:**
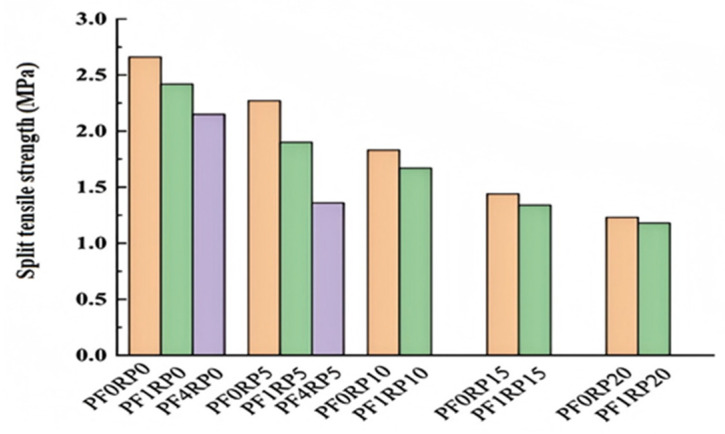
Effect of different fiber content and rubber content on splitting tensile strength [116].

The incorporation of PVAF exerts a complex influence on the internal structure and mechanical properties of RC, with its effect on tensile strength potentially leading to either an increase or decrease. According to the experimental findings of Naggar et al. [115], PVAF can effectively enhance the tensile strength of specimens containing TDA, with tensile strength improvements being directly correlated with increasing TDA content. For instance, the tensile strength of TDA80%–1%PVA specimens was 35% higher compared to TDA100%–1%PVA specimens, with a corresponding increase of 66%. In contrast, the study by Ma et al. [116] revealed a contrasting trend. Following the addition of PVAF, the tensile strength of RC experienced a further reduction. As illustrated in Figure 25, compared to specimens with a rubber content of 0–5%, the addition of PVAF caused a reduction in tensile strength by approximately 12.5%. In contrast, specimens with a 10–15% rubber content experienced a 7% reduction, while those with a 20% rubber content showed a reduced decrease of around 4%. A comparison of the splitting surfaces of the two specimen groups, before and after fiber addition, revealed that the shedding of rubber particles resulted in numerous voids within the RC. Following the addition of PVAF, both the quantity and diameter of these voids increased, which may contribute to the observed reduction in tensile strength.

#### 4.2.3. Flexural Strength

The incorporation of PVAF has demonstrated a substantial enhancement in the flexural strength of RC. Wang et al. [58] found that the flexural strength of concrete progressively diminished as the rubber particle content increases. Nevertheless, within a specific range of rubber content, the incorporation of PVAF mitigated the adverse impact of rubber on flexural strength, although it could not entirely negate this effect. In the FR-15 group specimens (comprising 15% rubber and 0.5% PVAF), while the flexural strength was lower than that of the control group, the reduction in flexural strength was less pronounced compared to the specimens without PVAF. Naggar et al. [115] demonstrated that the flexural strength of all specimens containing varying TDA contents was significantly enhanced following the incorporation of 1% PVAF. At TDA contents of 80% and 100%, the incorporation of PVAF resulted in an average increase in flexural strength of 35% and a maximum increase of 66%, respectively. The reinforcing effect of PVAF not only mitigated the weak bond between TDA and the cement matrix, but also provides substantial residual post-peak strength, thereby improving the deflection performance of specimens with a higher rubber content. In comparison to specimens without PVAF, those containing 1% PVAF were capable of withstanding greater loads before failure. Consequently, while the increase in the rubber particle content results in a reduction in flexural strength, the flexural properties of RC can be substantially improved through the addition of PVAF in optimal quantities.

#### 4.2.4. Durability Performance

The incorporation of PVAF significantly enhances the freeze–thaw resistance of RC. Wang et al. [58] demonstrated through experimental results that, during freeze–thaw cycles, the mass of ordinary concrete specimens gradually decreased. In contrast, specimens containing fibers and rubber, such as the FR-15, FR-20, and FR-25 groups, exhibited an increasing mass trend throughout the freeze–thaw process. Notably, the FR-25 group showed the highest mass increase, reaching 0.7%. However, after 225 freeze–thaw cycles, the mass of these specimens began to decline. These findings suggest that PVAF effectively mitigates damage expansion during freeze–thaw cycles and delays the onset of mass degradation.

**Figure 26:**
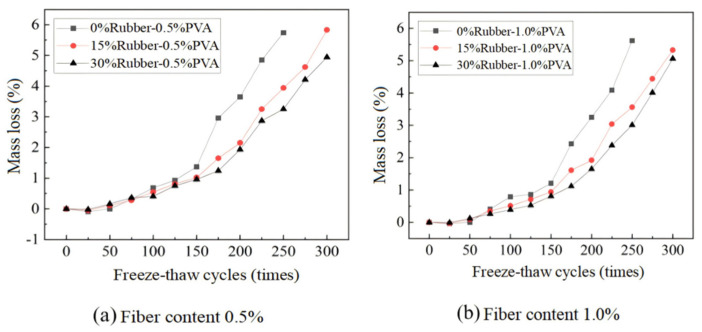
Line graph of mass loss rate and freeze–thaw cycle number at different fiber and rubber content [117].

Liu et al. [117] further corroborated these results, showing that RC without PVAF experienced significant mass loss during freeze–thaw cycles, while PVAF incorporation effectively reduced this deterioration. After 300 freeze–thaw cycles, concrete without PVAF exhibited substantial mass loss, whereas RC specimens containing 0.5% and 1.0% PVAF showed a markedly lower mass loss rate (Figure 26). Their study demonstrated that PVAF enhanced internal structural stability, effectively minimizing spalling and aggregate detachment caused by freeze–thaw cycles.

Beyond improving freeze–thaw resistance, PVAF also positively influences the drying shrinkage characteristics of RC. In the study conducted by Wang et al. [58], the specimen containing 0.5% PVAF (C-3) exhibited the least length change throughout the curing period. These findings suggest that the synergistic interaction between PVAF and rubber particles effectively suppresses deformation within the concrete matrix, reducing microcrack formation and propagation, thereby optimizing drying shrinkage behavior.

In conclusion, the incorporation of PVAF not only significantly enhances the freeze–thaw resistance of RC and mitigates mass loss during freeze–thaw cycles but also effectively alleviates the adverse effects of drying shrinkage induced by rubber particles. Through the synergistic interaction between PVAF and rubber particles, the overall performance and durability of concrete are significantly improved.

### 4.3. Polymer Hybrid Fiber Reinforced-Rubber Concrete

#### 4.3.1. Compressive Properties

**Figure 27:**
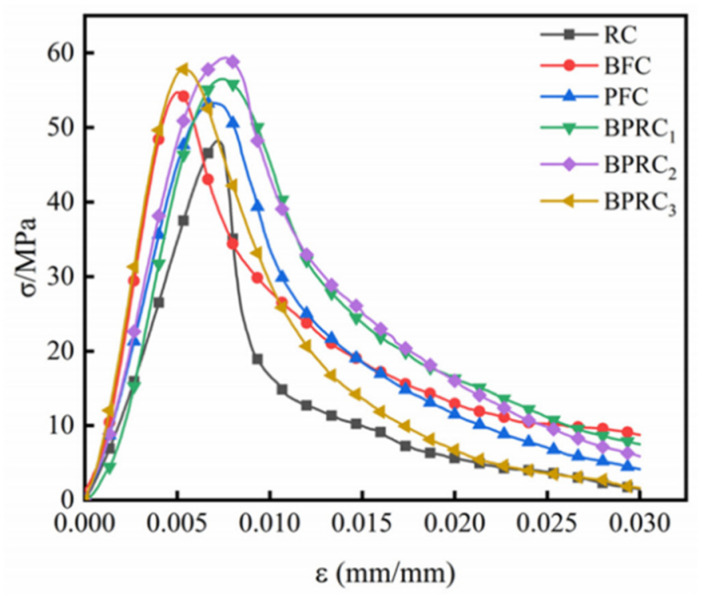
Uniaxial compression stress–strain curves of hybrid fiber RC [109].

To enhance the mechanical properties of RC, fiber incorporation is commonly employed. Numerous studies have shown that compared to using a single type of fiber, mixed fiber reinforcement yields more substantial performance improvements. Kun et al. [118] incorporated basalt fibers and PPF into RC to counteract the strength reduction caused by rubber particles. Their experimental results revealed that the compressive strength of RC with a basalt–polypropylene fiber mixture was 2.58% higher than that of the control group without PPF, demonstrating the significant strengthening effect of mixed fibers. Su et al. [109] conducted a similar study, investigating the impact of combining basalt fiber and PPF on RC’s mechanical properties through uniaxial compression tests. In their experiment, the rubber content and fine aggregate replacement ratio remained constant, while the volume ratio of basalt fiber to PPF was maintained at 1.5%. The test results indicated that mixed fibers effectively enhanced the compressive capacity of RC (Figure 27). The observed increase in peak stress further substantiates the superior performance of mixed fibers over single fibers in improving compressive strength. Liang et al. [119] found that combining 0.1% PPF with 0.9% steel fiber led to the most significant improvement in RC’s compressive strength. Although replacing 20% of fine aggregate with rubber particles substantially reduces compressive strength, incorporating a low content of polypropylene fiber alongside a high content of steel fiber effectively counteracts this reduction, yielding greater improvements than single fiber addition. This finding was further corroborated by Alwesabi et al. [120], who also demonstrated that the mixed addition of 0.1% PPF and 0.9% steel fiber significantly enhanced RC’s compressive strength.

In conclusion, hybrid fiber incorporation, particularly the combination of polypropylene, basalt, and steel fibers, significantly enhances the compressive strength and overall mechanical properties of RC. The synergistic effect of hybrid fibers more effectively improves structural integrity compared to individual fibers, particularly in mitigating the strength reduction caused by rubber particles.

#### 4.3.2. Splitting Tensile Strength

The incorporation of basalt fiber and PPF into RC effectively offsets the strength loss caused by rubber particles while enhancing splitting tensile strength. The synergistic effect of these two fibers surpasses that of individual fibers in improving RC’s mechanical properties. Kun et al. [118] conducted an orthogonal test to evaluate the mechanical properties of basal–-polypropylene fiber-reinforced rubber concrete at various dosages, analyzing the results comprehensively. The splitting tensile strength of basalt-polypropylene fiber-reinforced rubber concrete increases by 5.86% at a PPF content of 0.15% compared to the control group (without PPF). Similarly, with a basalt fiber content of 0.2%, the splitting tensile strength rises by 15.64% compared to the control (without basalt fiber), demonstrating the effectiveness of fiber incorporation. Alwesabi et al. [120] further validated these findings. Their study showed that adding 20% rubber particles to all concrete mixtures led to a reduction in splitting tensile strength. However, the mixture reinforced with 0.1% PPF and 0.9% steel fiber exhibited the highest splitting tensile strength among both hybrid and single fiber-reinforced rubber concretes, reaching 3.7 MPa. This hybrid fiber combination provided the most significant improvement.

Similarly, Qiu [121] emphasized through experimental results that hybrid fiber incorporation optimizes the strengthening effect of steel fibers while maximizing the benefits of PPF reinforcement during curing. When a 0.9% steel fiber and 0.1% PPF hybrid combination was used, the splitting tensile strength of RC reached 3.72 MPa, surpassing that of other hybrid fiber-reinforced rubber concrete mixtures.

#### 4.3.3. Impact Resistance

**Figure 28:**
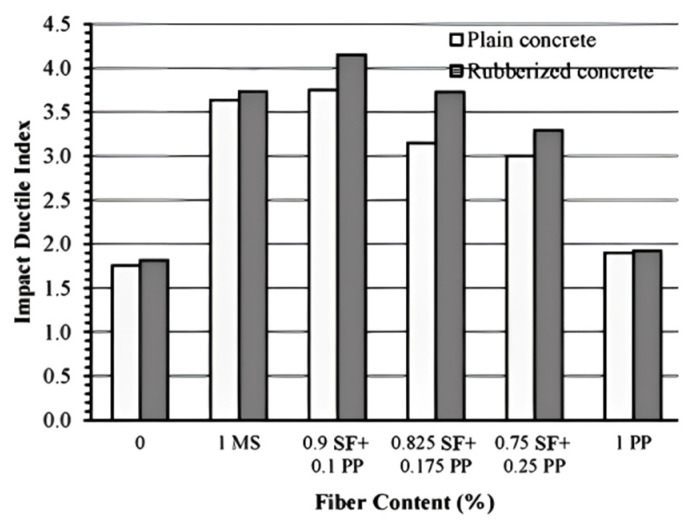
Impact ductility index of different concrete mixes [120].

Composite concrete reinforced with two or more fiber types benefits from the synergistic interaction between fibers, leading to superior performance compared to single fiber reinforcement. Steel fibers, with their high tensile strength and elastic modulus, effectively control the formation of initial cracks. Meanwhile, PPF, being soft and short, plays a crucial role in controlling the propagation of microcracks and small fissures while also enhancing the material’s ability to absorb impact energy. Alwesabi et al. [120] experimentally demonstrated that the interaction between steel and polypropylene hybrid fibers significantly enhanced the crack propagation resistance and impact resistance of RC. Their results showed that RC specimens reinforced with 0.9% steel fiber and 0.1% polypropylene fiber exhibited an ultimate impact energy of approximately 887.2 J—ten times greater than that of ordinary concrete. Additionally, the impact ductility index reached 4.15, doubling that of conventional concrete. The combination of rubber particles and hybrid fibers resulted in a favorable interaction, greatly improving impact resistance. Moreover, the incorporation of steel and polypropylene fibers effectively controlled both macro- and microcracks, further enhancing the durability of the concrete specimens. These findings highlight the strong resistance of steel–polypropylene fiber-reinforced rubber concrete to impact loads, underscoring its potential for impact-resistant applications. Notably, the hybrid fiber combination significantly improved both impact energy and the impact ductility index of RC, as illustrated in Figure 28. This enhancement can be attributed to the ability of hybrid fibers to control crack propagation, thereby slowing the rate of crack growth.

Therefore, concrete mixtures incorporating rubber particles and hybrid fibers demonstrate the highest ultimate impact energy. Steel–polypropylene fiber-reinforced rubber concrete proves to be an effective impact-resistant material, offering enhanced energy absorption and structural resilience.

#### 4.3.4. Durability Performance

**Figure 29:**
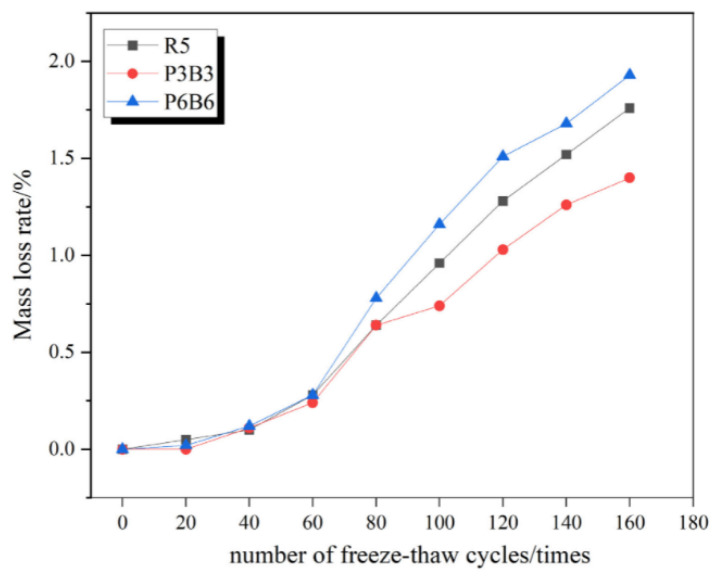
Mass loss rate curve in water [122].

Studies have demonstrated that the inclusion of hybrid fibers positively influences the durability of RC under freeze–thaw conditions, with the most notable improvements occurring at optimal fiber ratios. Tao et al. [122] conducted freeze–thaw cycle experiments on basalt–polypropylene fiber-reinforced rubber concrete and observed that while the mass loss rate of each specimen group increased with the number of freeze–thaw cycles, the P3B3 group (0.3% basalt fiber and 0.15% PPF, with 5% rubber content) exhibited a significantly lower mass loss rate compared to the other groups, as shown in Figure 29. After 160 freeze–thaw cycles, the mass loss rate of the R5 group (5% rubber content) was relatively high, while that of the P3B3 group remained comparatively low. This improvement is attributed to the fact that an appropriate fiber content contributes to increased density in the concrete, thereby reducing mass loss caused by the freezing and expansion of internal pore water.

### 4.4. Summary of PFRRC Performance Characteristics

The incorporation of an optimal amount of polymer fibers into RC can substantially enhance its mechanical properties. The inclusion of polymer fibers has been shown to increase the compressive strength of RC by up to 29%, the tensile strength by as much as 38%, and the flexural strength by as much as 66%. Notably, the performance improvements achieved with mixed polymer fibers are considerably more pronounced than those obtained with a single type of polymer fiber. Therefore, precise control over the type, ratio, and combination of polymer fibers is essential to optimize the mechanical properties of RC.

The inclusion of an optimal amount of polymer fibers can also substantially improve the durability of RC. Polymer fibers have been shown to significantly enhance the impact resistance of RC, with its final impact energy being up to 10 times higher than that of ordinary concrete. Furthermore, the incorporation of polymer fibers strengthens the internal cohesion of the RC matrix, mitigating issues such as peeling and detachment due to freeze–thaw cycles, and reducing mass loss by approximately 5%. Additionally, polymer fibers positively affect the drying shrinkage behavior of RC by inhibiting matrix deformation and limiting the formation and propagation of microcracks, thus improving the material’s overall shrinkage performance.

However, there remains limited research on the interactions between the content, aspect ratio, shape, and other characteristics of rubber particles and polymer fibers. This highlights the need for further investigation to fully understand their combined effects.

## 5. Conclusions

In RC, the incorporation of rubber particles substantially enhances the toughness and impact resistance of concrete. Under external forces, rubber particles effectively absorb energy through mechanisms such as surrounding microcracks and internal tensile cracking, with their fracture and impact failure energies increasing by up to 3.5 times that of conventional concrete. Furthermore, an appropriate rubber content can enhance the durability of RC, including resistance to chloride ion penetration, freeze–thaw cycles, and wear. For instance, due to its hydrophobic nature, rubber can create impermeable voids within the concrete, reducing the chloride ion diffusion coefficient by 24.5%. However, the incorporation of rubber particles diminishes the strength of RC, with the reduction being positively correlated to the rubber content, resulting in a maximum flexural strength reduction of 84%. To address this issue, researchers have suggested various modification techniques, including pretreatment of rubber particles and blending with materials such as silica fume and kaolin. These modification techniques facilitate enhanced adhesion between rubber particles and the concrete matrix, thereby improving compressive strength. Although these methods have yielded some results, a unified optimization standard remains absent. Determining how to precisely control modification parameters to achieve optimal performance improvements remains a significant challenge in current research.

In contrast, PFRC can improve both the mechanical and durability properties of concrete through the incorporation of an optimal amount of polymer fibers. Studies have demonstrated that the inclusion of an optimal amount of polymer fibers can increase the tensile strength of concrete by approximately 10–30% and reduce the chloride ion migration depth by a similar margin. Hybrid fibers further amplify impact resistance (ultimate impact energy up to 887 J, 10× higher than ordinary concrete) and durability. However, the impact of polymer fibers with varying types and aspect ratios on the strength enhancement of PFRC requires further investigation, and a standardized approach for optimizing polymer fiber parameters to meet specific requirements has yet to be established. Furthermore, the influence of various combinations of polymer fiber types on the durability of PFRC—such as resistance to chloride ion permeability, sulfate erosion, and freeze–thaw cycles—has yet to be clearly defined. This area urgently requires more in-depth research.

As a high-performance composite material, PFRRC integrates the superior properties of RC and PFRC, demonstrating remarkable mechanical properties and durability. When compared to RC, PFRRC can achieve a compressive strength increase of up to 29%, a tensile strength improvement of up to 38%, and a flexural strength enhancement of up to 66%. These exceptional properties render PFRRC highly promising for various engineering applications. However, existing studies have yet to conduct an in-depth exploration of the interactions between rubber and polymer fibers, the optimal content ratios, and other related aspects. Moreover, disagreements persist regarding the synergistic effects of their key components. Specifically, current research on PFRRC remains constrained by several key limitations, including the lack of comprehensive investigations into the interactions among critical factors such as rubber particle content, aspect ratio, shape, and polymer fiber characteristics. Furthermore, the performance of PFRRC in extreme environments—such as high temperature, high humidity, and severe corrosion—requires further investigation.

## 6. The Application Prospects of PFRRC

In the field of road engineering, PFRRC provides effective and cost-efficient design solutions for roadways and bridges. It significantly improves the wear resistance of road surfaces, offers superior impact resistance, reduces damage and maintenance frequency, and maintains surface integrity and stability under high-intensity impacts. Additionally, polymer fibers and rubber particles enhance the internal structure of the concrete, improve freeze–thaw resistance, mitigate freeze–thaw damage, and ensure long-term durability in cold climates. These properties make PFRRC an ideal choice for highway bridge decks in cold regions, where it helps minimize surface peeling, cracking, and other forms of deterioration, thereby extending the service life of bridge decks and offering high-quality solutions for road projects in such environments.

In the domain of protective and seismic engineering, the incorporation of rubber particles imparts excellent ductility and toughness to PFRRC, enabling it to effectively absorb impact forces. Consequently, this material can be utilized in anti-collision bridge piers, retaining walls, slope protection, and other structural applications for protective purposes. Furthermore, it can also serve as a damping material in earthquake-resistant structures. During an earthquake, it can dissipate seismic energy, reduce building vibrations and displacements, and mitigate structural damage.

Future research should prioritize determining the optimal proportions of various polymer fibers (e.g., PPF, PVAF, basalt fiber) and rubber in PFRRC, while further exploring their synergistic interactions to enhance compatibility and functional integration. This dual focus aims to identify the most effective fiber–rubber combinations and content ratios based on key performance criteria, such as mechanical strength, crack resistance, and durability. By elucidating how these components interact—whether through mutual reinforcement or property compensation—researchers can refine material designs tailored for specific engineering applications, such as seismic-resistant structures and high-wear pavement systems. Such advancements will not only maximize the material’s effectiveness but also broaden its applicability across diverse engineering fields, including civil infrastructure, transportation, and industrial flooring. In parallel, further exploration of novel fiber combinations, building upon existing mixed-fiber formulations, is essential to assess their impact on PFRRC performance. Additionally, the synergistic enhancement mechanisms between different fiber types should be investigated through a combination of microstructural analysis and macro-scale performance testing.

The development of PFRRC with improved overall performance represents a crucial direction for future research. Beyond enhancing mechanical properties, PFRRC contributes to sustainable construction by repurposing waste tires and reducing cement consumption, aligning with circular economy principles. To further minimize its environmental footprint, future innovations should explore bio-based fibers and advanced rubber treatments. Moreover, it is imperative to evaluate PFRRC’s performance under extreme environmental conditions, such as high temperatures, elevated humidity, and severe corrosion. Such investigations will facilitate the development of PFRRC materials tailored for these challenging conditions, thereby expanding their potential applications in demanding engineering environments.

In summary, PFRRC bridges material innovation and practical engineering needs, offering a versatile solution for resilient, sustainable infrastructure. Strategic research and standardization will drive its adoption across civil engineering domains.

## Figures and Tables

**Table 1:** Table of average compressive strength of coarse and fine aggregate replacement [21].

Type of Rubber	Percentage Replacement of Waste Tire Rubber (%)					
	5	10	20	30	40	50
	Relative Strength Crumb Rubber Concrete at Different Percentages					
	S.D.					
Coarse aggregate rep.	82.4 ± 6.5	69.9 ± 8.4	42.5 ± 10.5	33.5 ± 17.5	28.3 ± 13.3	31 ± 19.5
Fine aggregate rep.	87.1 ± 5.2	76 ± 7	59.5 ± 13.4	43.9 ± 14.9	32.3 ± 14.5	39.7 ± 27.8

**Table 2:** RC mechanical properties test results [45].

Batch No.	Compressive Strength (MPa)	Split Tensile Strength (MPa)	Modulus of Elasticity (MPa)	Poisson’s Ratio	Slump (cm)	Air Content (%)
1	39.08	3.11	34.72	0.23	14.7	4.5
2	22.33	2.29	28.89	0.22	14.4	4.1
3	23.23	2.19	28.88	0.21	14.3	5.0
4	22.89	2.38	28.69	0.22	14.2	4.6
5	22.16	2.17	26.93	0.23	14.6	4.8
6	20.48	2.15	28.59	0.25	15.2	5.0
7	20.82	1.99	28.39	0.24	14	4.7
8	22.83	2.41	30.4	0.25	14.8	4.6
9	25.14	2.62	31.19	0.21	15.2	4.9
10	25.6	2.69	31.96	0.23	14.8	5.0

**Table 3:** Fracture toughness of rubber concrete represented by K_IC_, G_IC_, J_IC_, and G_f_ [42].

Replacement Ratio	K_IC_ (MPa m^−1/2^)	G_IC_ (Nm/m^2^)	J_IC_ (Nm/m^2^)	G_f_ (Nm/m^2^)
Control	0.295	78.7	29.3	101.5
25%	0.601	281.0	68.1	300.6
50%	0.158	109.4	89.3	164.1
75%	0.149	130.4	133.0	162.6
100%	0.095	96.6	113.9	121.8

Note: G_f_: Fracture Energy, G_IC_: Critical energy release rate, K_IC_: Critical stress intensity, J_IC_: Toughness elastic modulus.

**Table 4:** Mechanical properties test results of specimens [74].

Specimens Code	P0	P1	P2	P3	P4
Strength (MPa)					
Compression (7 days)	39.4	42.3	41.7	38.4	39.2
Compression (28 days)	49.0	51.8	51.6	49.3	50.1
Splitting tensile	9.5	9.8	10.2	11.0	10.8
Flexural	8.7	9.2	8.9	9.7	10.2
Bending toughness (kg·m)	0.73	1.22	2.31	2.53	1.56
toughness index I_5_	-	1.29	1.28	1.25	1.28
toughness index I_10_	-	-	1.52	1.47	1.03
toughness index I_30_	-	-	-	1.68	-
Water infusion (mm)	14.2	14	13.5	13.4	14.03
Air permeability (m^2^)	6.68	6.60	6.52	6.48	6.63

## Data Availability

No new data were created or analyzed in this study.
